# Comparative efficacy of Honghua class injections for treating acute ischemic stroke: A Bayesian network meta-analysis of randomized controlled trials

**DOI:** 10.3389/fphar.2022.1010533

**Published:** 2022-09-28

**Authors:** Lan Li, Chongyu Shao, Zheting Liu, Xiaolong Wu, Jiehong Yang, Haitong Wan

**Affiliations:** ^1^ College of Life Science, Zhejiang Chinese Medical University, Hangzhou, China; ^2^ College of Basic Medical Sciences, Hangzhou, China

**Keywords:** Honghua class injections, acute ischemic stroke, network meta-analysis, Bayesian, randomized controlled trials, comparative efficacy

## Abstract

**Background:** Acute ischemic stroke (AIS) is associated with high morbidity, mortality, and disability. Clinical trials have shown that Honghua class injections (HCIs) combined with WM achieve better clinical efficacy than WM alone. In this study, we performed a Bayesian network meta-analysis (NMA) of randomized controlled trials (RCTs) to evaluate the efficacy of different HCIs combined with WM in treating AIS.

**Methods:** First, the inclusion and exclusion criteria were established. From inception to 1 June 2022, a systematic literature search was conducted in multiple databases for the treatment of AIS with HCIs, including Honghua injection (HI), Safflower Yellow injection (SYI), Guhong injection (GHI), and Danhong injection (DHI). Subsequently, OpenBUGS 3.2.3 was applied to conduct a Bayesian algorithm, and Stata 16.0 was used to prepare the graphs. Multidimensional cluster analysis was performed using the “scatterplot3d” package in R 3.6.1 software.

**Results:** In this NMA, a total of 120 eligible RCTs were included, involving 12,658 patients, and evaluating the clinical effectiveness rates, activities of daily living (ADL), hemorheological indexes, and adverse reactions (ADRs). DHI + WM was the best intervention for improving the clinical effectiveness rate. Moreover, cluster analysis demonstrated that DHI + WM and SYI + WM had better comprehensive therapeutic effects. As most of the included RCTs did not monitor ADRs, the safety of the HCIs remains to be further explored.

**Conclusion:** DHI + WM and SYI + WM probably have a better clinical efficacy on AIS patients. Nevertheless, due to the limitation of this NMA, this conclusion may be biased. High-quality RCTs should be performed to validate our findings.

**Systematic Review Registration:** https://www.crd.york.ac.uk/PROSPERO/, identifier CRD42021229599

## 1 Introduction

Stroke is the leading cause of death in China and the second-most prevalent cause of death worldwide (Benjamin et al., 2018; Zhou et al., 2019). The prevalence of stroke in China has been increasing continuously since 2006, with ∼13 million stroke patients. Ischemic stroke (IS) has the highest incidence, accounting for ∼80% of all stroke patients (Benjamin et al., 2018). Due to its high morbidity, mortality, and disability, stroke has become a major disease that seriously endangers human health. At present, the treatment of acute ischemic stroke (AIS) mainly includes thrombolysis, intervention, anti-platelet aggregation, anticoagulation, lowering the levels of fibrinogen and blood lipids, expansion of blood capacity, and neuroprotection. Notably, the most effective treatment is ultra-early thrombolysis (Ospel et al., 2020). However, due to the strict treatment time window of thrombolysis, the population in which this treatment is carried out is extremely limited in China (Zhu et al., 2020). Therefore, it is necessary to explore other effective therapeutic methods. Traditional Chinese medicine (TCM) has several advantages, such as multiple targets, good synergy, and low side effects, widely used in treating complex diseases (Chen et al., 2017). TCM-related injections (TCMIs) are often combined with western medicine (WM) for the treatment of acute diseases in Chinese clinics and show instant effectiveness and high bioavailability (Li et al., 2017). The detailed pharmacology information, composition and the extraction procedure of HCIs have been stated unambiguously, shown in Supplementary Material S1.

In TCM theory, AIS is usually related to blood stasis syndrome and its treatment strategy involves the promotion of blood circulation and removal of blood stasis (Tang et al., 2012; Wang et al., 2020b). Honghua (*Asteraceae, Carthamus, Carthamus tinctorius L.*) is commonly used in the treatment of ischemic cardiovascular and cerebrovascular diseases, as it has a good effect on promoting blood circulation and removing blood stasis, which was described in the Compendium of Materia Medica (Ming Dynasty, ∼500 years ago) (Cao et al., 2014; Bai et al., 2020). Moreover, Hydroxysafflor yellow A is the main active component of Honghua (Bai et al., 2020). TCMIs contain an extract from Honghua, including Honghua injection (HI), Safflower Yellow injection (SYI), Guhong injection (GHI), and Danhong injection (DHI). Herein, we call them Honghua class injections (HCIs). All HCIs have been approved by the State Pharmaceutical Administration of China. HCIs combined with WM are commonly adopted in treating AIS and achieve a good clinical effect (Li et al., 2015b; Du et al., 2018; Feng et al., 2018).

Compared with traditional meta-analyses, network meta-analysis (NMA) can synthesize multiple interventions and perform direct or indirect comparisons for the same disease (Guo et al., 2020). Moreover, it can help to evaluate and rank the efficacy of different treatments (Catalá-López et al., 2014). Existing studies demonstrated that HCIs combined with WM achieved better clinical efficacy than WM alone in the treatment of AIS. However, there is a lack of clinical trials comparing HCIs directly. Therefore, it is necessary to evaluate and compare the efficacy of various HCIs by NMA. In this study, four HCIs, including HI, SYI, GHI, and DHI were selected as adjuvant therapies for AIS, which were all combined with conventional WM treatments. Subsequently, we applied a Bayesian NMA to explore the comparative effectiveness and safety between different HCIs combined with WM against AIS, providing a reference for clinical practice.

## 2 Methods

This NMA has been registered on the International Prospective Register of Systematic Review (PROSPERO) platform (CRD42021229599). This NMA study was conducted strictly according to the guidelines based on the Preferred Reporting Items for Systematic Reviews and Meta-Analyses (PRISMA) as shown in Supplementary Material S2 (Moher et al., 2009).

### 2.1 Search strategy

All RCTs focusing on HCIs against AIS literatures were searched electronically from the following seven databases, Cochrane Library, PubMed, China National Knowledge Infrastructure Database (CNKI), China Biomedical Literature Service System (SinoMed), and Wan-Fang Database. All database searches were conducted on studies dating from inception to 1 June 2022, with no restrictions on language. The Medical Subject Heading (MeSH) terms and free-text keywords were utilized, including “acute ischemic stroke (MeSH Terms),” “ischemic stroke,” “acute ischemic stroke,” “stoke,” “brain infarction,” “acute cerebral infarction,” “cerebral infarction,” “brain embolism,” “cerebrovascular disorders,” “Honghua injection,” “Safflower Yellow injection,” “Guhong injection,” “Danhong injection,” and “randomized controlled trial (Publication Type).” In addition, there were no restrictions on the blinding methods, publication year, and language. Furthermore, the specific retrieval strategy is shown in Supplementary Material S3.

### 2.2 Inclusion criteria

#### 2.2.1 Patient populations

All included cases were diagnosed with AIS. This study only recruited patients within 2 weeks of onset. There were no restrictions on the age, gender, race, and severity of disease.

#### 2.2.2 Interventions and comparators

The interventions of experiment groups were HCIs (HI, SYI, GHI, or DHI) combined with WM treatments. The control group only received WM therapy. Conventional WM treatment, including anti-platelet aggregation; anticoagulation; lipid-lowering; correction of water, electrolyte disorders, and acid-base imbalance; improvement of cerebral circulation and using neuroprotective agent. The dosage and duration of treatment was not restricted.

#### 2.2.3 Outcomes

The primary outcome was the clinical effectiveness rate, according to the National Institute of Health Stroke Scale (NIHSS) evaluation: a reduction of 91%–100%, 46%–90%, and 18%–45% corresponds to “basic cure,” “notable progress,” and “progress” respectively, which defined the effectiveness (Wang et al., 2018).

The secondary outcomes were the activities of daily living (ADLs), using Barthel Index Scale Scores; hemorheological indexes, including low shear blood viscosity (LBV), high shear blood viscosity (HBV), plasma viscosity (PV), fibrinogen (FIB) levels; and adverse reactions (ADRs), including adverse drug events (ADEs).

#### 2.2.4 Study design

Randomized controlled trials (RCTs) of HCIs combined with WM in the treatment of AIS. RCTs were not restricted by language, country, publication date, or stage.

### 2.3 Exclusion criteria

The RCTs that met one of the following conditions were excluded: 1) RCTs that did not meet the criteria for clinical efficacy evaluation; 2) thrombolytic therapy; 3) interventions involving a combination therapy with Chinese herbal medicine, acupuncture or other TCM injections; 4) the full text of the study was unavailable; 5) incorrect or incomplete data; 6) duplicate reports; and 7) no related outcomes.

### 2.4 Data extraction

All the articles were managed by NoteExpress software (Tongji University Library, Shanghai, China) and selected by two independent reviewers by excluding irrelevant articles, reviews, and animal experiments. Two reviewers independently extracted the eligible research data using Excel (Microsoft, United States) and the collected the information as follows: 1) sample size in each group, sex, age, and duration of disease; 2) intervention (the types of HCIs, dose, and course of treatment); 3) outcome indicators: clinical effectiveness rate, ADL, LBV, HBV, PV, FIB levels, and ADRs/ADEs; 4) factors to evaluate the risk of bias.

### 2.5 Quality assessment

In this study, we applied the Cochrane bias risk assessment tool to assess the methodological quality of the included RCTs (Higgins et al., 2011). The tool assessment items were as follows: random sequence generation, allocation concealment, blinding of participants and researchers, blinding of outcome assessment, incomplete outcome data, selective reporting, and other bias. There were two independent investigators (LL and CS) who assessed the quality of the included RCTs. Any disagreement was resolved by a third researcher (HW) or by consensus. RevMan 5.4 software was applied to generate the risk of bias diagram for quality evaluation.

### 2.6 Statistical analysis

Bayesian modeling was performed using OpenBUGS 3.2.3, (MRC Biostatistics Unit, Cambridge, UK) (Owen et al., 2020). Each chain in the OpenBUGS program has 50,000 iterations, with the first 20,000 iterations being burn-in tests to remove the initial value effect. The Odds ratio (OR) and mean difference (MD) with a 95% credible interval (CI) were used to estimate the binary results and continuous data, respectively (Da Costa et al., 2013; Pateras et al., 2018). If the ORs did not include 1 and MDs did not cover 0, the difference between the two groups was deemed significant (Da Costa et al., 2013; Pateras et al., 2018).

The software, Stata 16.0 (College Station, TX, United States) was applied to generate graphs, carry out the publication bias test, and consistency test. In the network graphs, nodes represented interventions, and the link lines indicated direct comparisons. For each outcome, the probability of the intervention was ranked using the surface under the cumulative ranking area (SUCRA), a larger area under the curve indicated a better cure (Zhou et al., 2020). Based on SUCRA value, R 3.6.1 software (Mathsoft, Cambridge, United States) was applied to perform the cluster analysis using K-means method (Guo et al., 2020). In addition, “scatterplot3d” package was used for multidimensional cluster analysis. The comprehensive curative effects of HCIs in two or three outcomes were evaluated.

The publication bias was assessed using a comparison-adjusted funnel plot (Song et al., 2010). Publication bias did not exist if the funnel plots were symmetrical (Debray et al., 2018). Moreover, as this NMA had no closed loops, the overall consistency test could not be performed. However, a local consistency test was performed. It was determined that there were no local inconsistencies in this study when *p* > 0.05.

## 3 Results

### 3.1 Literature selection

**FIGURE 1:**
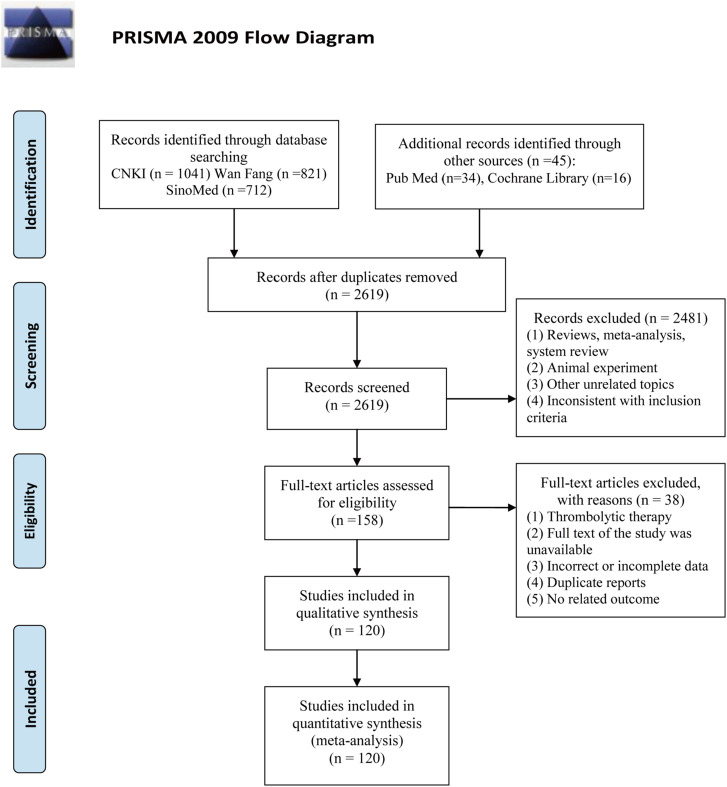
Flow chart for searching eligible studies.

Using the search strategy, a total of 2,669 articles were retrieved. After removing duplicates, 2,619 articles were obtained. Subsequently, 2,481 articles were excluded on account of being reviews, meta-analyses, systematic reviews, animal experiments, and other irrelevant literature, or on account of having inconsistencies in inclusion criteria. A total of 158 articles were evaluated for qualification. Next, 38 articles were excluded for the following reasons: thrombolytic therapy, full text of the study was unavailable, incorrect or incomplete data, duplicate reports, and no related outcomes. Finally, 120 RCTs were eligible for inclusion in this NMA (Figure 1).

### 3.2 Study characteristics

**FIGURE 2:**
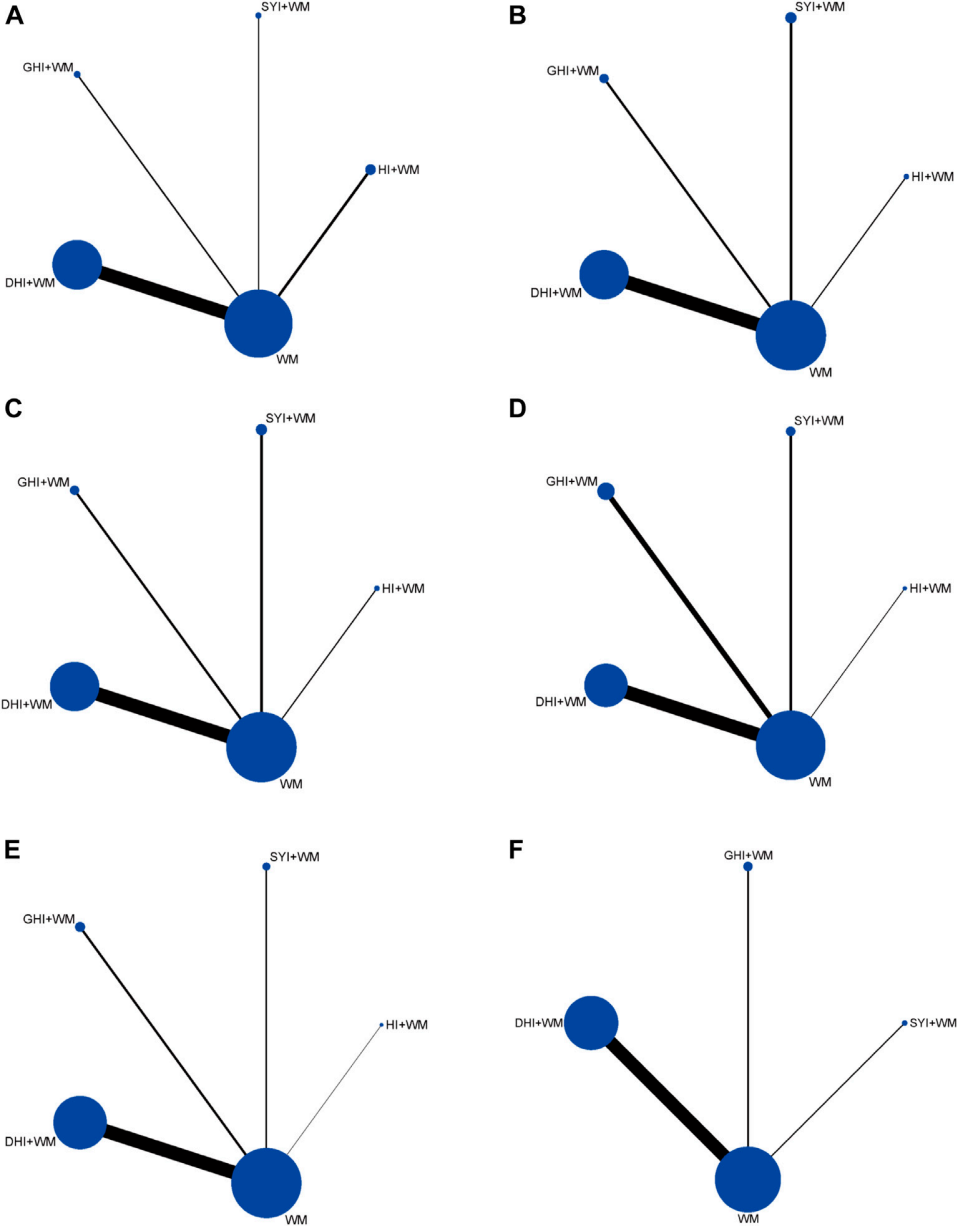
Network diagrams of the outcomes. **(A)** Clinical effectiveness rate; **(B)** ADL function; **(C)** LBV; **(D)** HBV; **(E)** PV; and **(F)** FIB.

Four types of HCIs were incorporated, including HI, SYI, GHI, and DHI. Overall, the 120 RCTs involved 12,658 patients (6,185 in the control group and 6,473 in the experiment group). A total of four comparisons were evaluated: HI + WM vs. WM (*n* = 15), SYI + WM vs. WM (*n* = 8), GHI + WM vs. WM (*n* = 12), and DHI + WM vs. WM (*n* = 85). The control groups were treated with WM, which primarily contained aspirin, anticoagulants, neuroprotectants, etc. The characteristic details of the RCTs were shown in Table 1, and the included literatures are described in Supplementary Material S4. The network graphs of the four HCIs with different outcomes were shown in Figure 2.

**TABLE 1:** Characteristics details of the studies NMA.

Study ID	Sample size		Sex(M/F)		Average age		Age range		Therapy		Course	outcomes
	C	E	C	E	C	E	C	E	E	C		
Jia, 2021	49	50	26/23	25/25	62.15 ± 8.26	61.86 ± 8.06	50∼78	48∼78	WM	HI + WM	14 d	②
Wang, 2018	30	30	NR		NR		NR		WM	HI + WM	14 d	①
Li, 2016	116	117	69/47	67/50	65.37 ± 1.24	64.35 ± 1.02	38∼80	37∼79	WM	HI + WM	NR	①⑦
Zhang, 2013	25	25	13/12	12/13	74.6 ± 7.1	76.3 ± 6.7	65∼82	68∼83	WM	HI + WM	28 d	①⑦
Liu, 2013	30	30	15/15	14/16	63 ± 13	64 ± 13	NR		WM	HI + WM	14 d	①
Li., 2012	60	60	81/39		48.32 ± 10.56		32∼63		WM	HI + WM	10 d	①
Liu, 2012	70	66	38/32	36/30	61.5	63.4	38∼80	43∼79	WM	HI + WM	14 d	①
Zhao, 2011	68	68	39/29	37/31	62.1	61.8	43∼82	41∼83	WM	HI + WM	14 d	①
Ge, 2011	44	62	37/25	27/17	NR		48∼78	52∼83	WM	HI + WM	20 d	①⑦
Lian, 2010	100	100	80/20	78/22	58.1	57.6	44∼67	45∼71	WM	HI + WM	28 d	①
Li, 2010	28	28	15/13	18/10	51.7 ± 10.9	52.3 ± 11.1	41∼69		WM	HI + WM	15-20 d	①
Xu, 2005	50	50	32/18	28/22	64 ± 10	59 ± 10	51∼68	49∼75	WM	HI + WM	14 d	①⑦
Lv, 2005	74	76	39/35	40/36	65.9 ± 8.1	66.4 ± 8.3	51∼70	50∼73	WM	HI + WM	15 d	①
Liu 2004	55	55	31/24	29/26	NR		49∼75	48∼73	WM	HI + WM	21 d	①③④⑤⑦
Song, 2002	30	45	16/14	27/18	59.64	60.5	38∼73	40∼81	WM	HI + WM	14 d	①②⑦
Song, 2021	68	68	73/63		64.7 ± 10.01		49∼82		WM	SYI + WM	14 d	①②⑦
Jiang, 2022	82	82	50/32	45/37	71.18 ± 3.29	71.12 ± 3.22	NR		WM	SYI + WM	14 d	①②③④⑥
Tang 2013	46	46	23/23	24/22	51.6 ± 10.4	52.1 ± 9.5	43∼73	45∼72	WM	SYI + WM	14 d	①⑦
Cheng 2019	60	60	40/20	41/19	63.23 ± 5.23	63.26 ± 5.21	44∼79	45∼78	WM	SYI + WM	14 d	①②③④⑤⑦
Guo 2016	40	40	25/15	26/14	63.5 ± 15.2	61.4 ± 13.6	45∼82	42∼80	WM	SYI + WM	14 d	①②⑦
Ji, 2016	48	48	26/22	25/23	63.32 ± 5.51	63.26 ± 5.47	50∼72	51∼73	WM	SYI + WM	14 d	①②⑤
Huang, 2011	63	63	42/21	44/19	53.2	52.8	42∼82	43∼80	WM	SYI + WM	14 d	①⑦
Li 2015	31	66	9/21	28/38	58.84 ± 10.2	59.47 ± 10.9	30∼70	31∼70	WM	SYI + WM	14 d	③④⑤⑥
Chen 2022	42	42	22/20	24/18	62.53 ± 1.24	62.63 ± 2.13	46∼78	46∼80	WM	GHI + WM	14 d	②
Song, 2021	40	40	17/13	26/14	63.37 ± 5.58	63.72 ± 6.03	49∼71	50∼72	WM	GHI + WM	14 d	③④⑤⑥
Zhao 2021	77	77	37/40	43/34	59.37 ± 4.56	61.13 ± 4.90	40∼80	41∼78	WM	GHI + WM	14 d	①③④⑤⑥⑦
Lu 2022	52	52	28/24	29/23	57.45 ± 5.98	57.33 ± 5.90	37∼75	37∼75	WM	GHI + WM	14 d	①②⑤⑦
Xiao, 2020	45	45	24/21	25/20	68.14 ± 2.32	69.15 ± 2.44	58∼75	60∼75	WM	GHI + WM	14 d	①②⑦
Jiang, 2016	40	40	20/20	19/21	NR		41∼75	40∼75	WM	GHI + WM	14 d	③④⑤
Sheng, 2019	38	38	23/15	21/17	63.24 ± 9.48	64.08 ± 9.16	45∼82	46∼82	WM	GHI + WM	14 d	①③④⑦
Li, 2018	30	30	18/12	18/12	63.1 ± 5.2	63.5 ± 5.3	46∼80	46∼80	WM	GHI + WM	10-15 d	①③④⑦
Li, 2018	68	68	41/27	39/29	61.9 ± 7.2		46∼83	48∼85	WM	GHI + WM	14 d	①②③④
Hu 2009	52	60	27/25	32/28	60.5 ± 7.3	60.8 ± 7.2	47∼76	45∼78	WM	GHI + WM	14 d	①③⑥⑦
Tang 2016	39	39	21/18	20/19	62.75 ± 5.58	62.35 ± 5.53	53∼75	52∼71	WM	GHI + WM	14 d	①③⑥
Zhang, 2010	220	239	143/77	138/101	61.17 ± 11.68	62.22 ± 10.22	NR		WM	GHI + WM	21 d	⑦
Wu, 2022	30	31	18/12	21/10	50.27 ± 4.63	50.25 ± 4.78	45∼75		WM	DHI + WM	14 d	①⑤⑦
He, 2021	30	30	19/11	20/10	68.61 ± 7.96	67.09 ± 8.57	54∼80	55∼82	WM	DHI + WM	14 d	①③④⑥
Wang 2021	68	68	46/22	44/24	60.15 ± 5.33	61.28 ± 5.29	48∼94		WM	DHI + WM	14 d	①②
Shen, 2021	50	50	23/27	22/28	65.42 ± 5.87	65.39 ± 5.59	53∼83	52∼82	WM	DHI+WM	14 d	①②
Zhang 2021	43	43	24/19	22/21	72.17 ± 3.42	72.43 ± 3.58	60∼88	60∼88	WM	DHI+WM	14 d	②⑦
Wang 2021	45	45	33/12	31/14	62.8 ± 4.5	62.3 ± 4.6	41∼70	42∼70	WM	DHI+WM	14 d	①②⑥
Yuan 2018	40	40	24/16	23/17	51.6 ± 2.5	52.3 ± 3.2	25∼80	24∼80	WM	DHI+WM	14 d	①②
Yuan, 2019	38	38	20/18	21/17	65.39 ± 2.19	65.50 ± 2.31	51∼73	52∼74	WM	DHI+WM	14 d	①②⑥⑦
Fan 2019	68	68	41/27	37/31	58.31 ± 10.1	60.04 ± 10.5	44∼76	46∼78	WM	DHI+WM	21 d	①②⑦
Wu, 2019	42	42	27/15	25/17	66.1 ± 7.3	66.8 ±7.1	48∼82	49∼84	WM	DHI+WM	14 d	①②③④⑤⑥
Li 2020	52	52	26/26	29/23	68.58 ± 4.68	68.34 ± 5.64	60∼79	60∼79	WM	DHI+WM	14 d	①⑤⑥⑦
Tang 2019	45	41	23/22	21/20	46.5 ± 7.9	48.7 ± 8.3	37∼73	38∼76	WM	DHI+WM	14 d	①②⑦
Zhang 2018	54	54	29/25	28/26	60.2 ± 3.1	61.8 ± 3.2	49∼72	51∼74	WM	DHI+WM	14 d	①⑤⑥
Kang 2020	60	65	35/25	37/28	54.95 ± 5.9	55.4 ± 5.99	41∼79	40∼81	WM	DHI+WM	14 d	②
Chen, 2019	165	165	86/79	83/82	59.8 ± 6.7	60.1 ± 6.2	36∼72	37∼73	WM	DHI+WM	15 d	①②⑥
Liu, 2019	49	49	24/25	25/24	59.32 ± 12.1	58.91 ± 12.2	NR		WM	DHI+WM	NR	①③④⑤⑥
Liu 2020	71	71	41/30	43/28	61.97 ± 8.32	63.24 ± 6.57	43∼75	47∼78	WM	DHI+WM	14 d	①②
Zhu 2020	30	30	16/14	18/12	57.62 ± 4.87	56.34 ± 5.29	NR		WM	DHI+WM	14 d	①②
Dai, 2018	47	47	22/25	26/21	56.43 ± 2.42	56.42 ± 2.43	36∼79	36∼78	WM	DHI+WM	15 d	①②⑦
Qiao, 2010	30	30	20/10	19/11	NR		43∼80	44∼79	WM	DHI+WM	14 d	①
Zhou, 2009	50	50	35/15	36/14	NR		44∼77	45∼78	WM	DHI+WM	14 d	①
Gu 2012	80	80	41/39	38/42	59.24	60.31	NR		WM	DHI+WM	15 d	①③④⑤⑥⑦
Zhang, 2010	60	60	40/20	38/22	69.3	68.9	42∼80	43∼79	WM	DHI+WM	14 d	①⑦
Wang 2017	50	50	52/48		53.6		41∼78		WM	DHI+WM	14 d	①⑦
Tan 2016	43	43	49/37		NR		39∼82		WM	DHI+WM	14 d	①⑦
Liu 2010	40	40	23/17	22/18	NR		51∼57	52∼70	WM	DHI+WM	15 d	①
Yi 2010	40	40	28/12	24/16	57 ± 7.5	55 ± 6.5	44∼78	45∼76	WM	DHI+WM	14 d	⑤⑦
Pang 2011	80	85	NR		NR		45∼78		WM	DHI+WM	14 d	①⑦
Jiang 2008	40	40	24/16	23/17	59.5 ± 11.75	59.8 ± 9.45	35∼85	36∼86	WM	DHI+WM	14 d	①⑦
Wang, 2008	30	30	14/16	17/13	64.1	63.7	49∼83	50∼85	WM	DHI+WM	15 d	③④⑤⑦
Hu 2008	32	32	21/11	18/14	59.1 ± 9.7	62.1 ± 5.7	56∼75		WM	DHI+WM	14 d	③④⑤⑥
Zheng 2015	84	84	51/33	53/31	50.21 ± 8.66	49.67 ± 8.27	33∼69	36∼68	WM	DHI+WM	14 d	①⑤⑥
Yun 2017	31	31	20/11	17/14	68.2 ± 7.1	69.5±7.3		58∼80	WM	DHI+WM	14 d	③④⑤⑥⑦
Wang, 2013	36	34	22/14	21/13	56.6 ± 7.4	56.3 ± 7.2	36∼84	34∼85	WM	DHI+WM	14 d	①⑦
An 2015	35	35	22/13	20/15	57 ± 15	59 ± 15	43∼78	45∼80	WM	DHI+WM	14 d	①
Li, 2017	30	30	18/12	19/11	62.03 ± 4.11	61.48 ± 3.95	42∼70	41∼71	WM	DHI+WM	14 d	①⑥⑦
Zhang 2016	40	40	48/32		67.5		NR		WM	DHI+WM	14 d	①③④⑤⑥⑦
Yan, 2013	59	57	32/27	31/26	NR		41∼73	43∼72	WM	DHI+WM	14 d	①⑦
Gao, 2011	30	32	16/14	17/15	65.5	63.2	45∼76	40∼79	WM	DHI+WM	14 d	①
Zhang, 2012	57	64	38/19	43/21	69.7 ± 12.8	71.4 ± 11.3	51∼77	52∼79	WM	DHI+WM	14 d	①
Huang, 2017	57	63	72/68		64.37±1.56	64.61 ± 2.34	NR		WM	DHI+WM	28 d	①⑦
Li, 2014	32	32	20/12	22/10	52 ± 9.8	54 ± 10.1	43∼68	45∼70	WM	DHI+WM	14 d	①⑦
Wu 2011	40	40	18/14	20/12	62 ± 7	62 ± 5	NR		WM	DHI+WM	28 d	①
Ma, 2017	42	40	27/15	24/16	65.26 ± 7.23	60.01 ± 7.86	49∼75	39∼70	WM	DHI+WM	14 d	①⑥⑦
Guan 2017	40	40	26/14	25/15	58.79 ± 5.78	59.83 ± 5.16	41∼75	43∼74	WM	DHI+WM	14 d	①③④⑤⑦
Ma 2012	50	50	29/21	28/22	62.2 ± 8.1	61.3 ± 7.9	42∼81	41∼79	WM	DHI+WM	14 d	①
Xiao, 2016	35	35	21/14	23/12	59.0 ± 9.7	59.0 ± 9.7	NR		WM	DHI+WM	30 d	①②③④⑤⑥⑦
Yang 2017	42	42	27/15	26/16	52.2 ± 5.2	52.9 ± 5.1	46∼72	47∼72	WM	DHI+WM	14 d	①⑥
Zhang, 2017	45	45	26/19	24/21	69.3 ± 9.6	68.9 ± 9.5	60∼85	59∼84	WM	DHI+WM	14 d	①⑤
Zeng 2016	55	55	31/24	30/25	63.3 ± 5.3	63.0 ± 5.2	38∼74	38∼74	WM	DHI+WM	15 d	①②
Mao, 2010	29	29	18/11	17/12	61.1	61.3	43∼73	45∼75	WM	DHI+WM	14 d	①
Zhang, 2015	90	90	88/92		51.2 ± 5.4		45∼67		WM	DHI+WM	14 d	①⑦
Li, 2011	32	32	19/13	22/10	67.3	65.8	43∼79	42∼77	WM	DHI+WM	14 d	⑦
Shi, 2010	40	43	28/12	29/14	64	65	41∼78	42∼79	WM	DHI+WM	14 d	①⑦
Zhao 2010	96	96	53/43	59/37	65.60 ± 3.44	67.40 ± 3.48	53∼79	52∼80	WM	DHI+WM	14 d	①②④⑥
Cai 2009	100	120	64/36	76/44	59.8	58.7	40∼74	41∼72	WM	DHI+WM	30 d	①③④⑤⑥
Wang 2012	40	80	28/12	52/28	63.6 ± 11.2	64.7 ± 10.5	43∼83	45∼81	WM	DHI+WM	20 d	①⑦
Zhang 2011	60	60	36/24	38/22	63.4 ± 13.18	62.4 ± 11.06	42∼83	40∼81	WM	DHI+WM		①③④⑤⑥
Chen, 2008	54	52	36/18	38/14	65.7 ± 7.39	63.0 ± 6.7	51∼75	50∼72	WM	DHI+WM	14 d	①⑦
Huang, 2010	76	76	47/29	49/27	62.0 ± 2.5	63.5 ± 3.5	NR		WM	DHI+WM	14 d	①⑤⑥
Zhou, 2007	30	30	20/10	19/11	NR		43∼80	44∼79	WM	DHI+WM	14 d	①
Fan 2018	35	35	NR		50.45 ± 9.56	49.32 ± 9.37	32∼78	34∼76	WM	DHI+WM	14 d	①②
Lv, 2017	42	42	28/14	25/17	58. 8 ± 8. 7	59. 2 ± 8. 6	42∼79	40∼78	WM	DHI+WM	14 d	①②
Liu, 2015	40	40	45/35		66.3 ± 1.4		50∼88		WM	DHI+WM	14 d	①
Zhang 2012	60	60	31/29	32/28	64.0 ± 12.3	63.3 ± 11.5	28∼83	32∼81	WM	WM	14 d	②
Yue, 2012	30	30	18/12	19/11	NR		40∼77	41∼80	WM	WM	15 d	①⑤
Wu, 2012	46	48	27/19	28/20	64.8 ± 11.7	65.3 ± 12.6	52∼80	54∼81	WM	WM	14 d	①⑦
Jiang, 2011	28	31	13/15	15/16	NR		50∼78	51∼79	WM	WM	14 d	①⑦
Li 2012	32	36	17/15	20/16	65.2	63.5	52∼76	51∼78	WM	WM	14 d	①⑥⑦
Su 2012	37	38	21/16	22/16	63.01 ± 9.34	61.58 ± 9.17	45∼80	42∼85	WM	WM	14 d	①③④⑤⑥⑦
Chen 2013	64	70	30/34	37/33	NR		44∼78	43∼79	WM	WM	14 d	①
Wang 2010	80	80	44/36	46/34	65.75 ± 6.86	66.36 ± 5.73	52∼78	55∼80	WM	WM	14 d	②③④⑤
Gao, 2011	30	32	16/14	17/15	65.5	63.2	45∼76	40∼79	WM	WM	14 d	①
Han, 2016	50	50	30/20	29/31	70.1 ± 2.4	70.3 ± 2.6	55∼80	54∼80	WM	WM	14 d	①
Fang 2013	48	48	50/46		50.6 ± 5.9		52∼64		WM	WM	14 d	①⑦
Li 2017	40	40	43/37		66.5 ± 10.2		50∼77		WM	WM	14 d	①②⑦
Zhong, 2011	35	35	33/37		72 ± 5		61∼78		WM	WM	14 d	①
Fan, 2016	52	115	27/26	59/56	54.8 ± 3.0	54.7 ± 3.1	50∼77	48∼77	WM	WM	14 d	①⑦
Xu, 2014	45	45	24/21	25/20	70.9 ± 1.2	71.3 ± 1.1	57∼79	56∼78	WM	WM	14 d	①
Liu 2018	47	47	30/17	29/18	64.83 ± 6.24	64.75 ± 6.19	54∼82	53∼81	WM	WM	14 d	①②⑦
Tao, 2011	20	55	12/8	34/21	55.4 ± 2.3	56.5	42∼76	43∼78	WM	WM	15 d	①⑤⑥⑦
Jiang 2016	54	54	32/22	30/24	60.96 ± 9.19	61.78 ± 8.36	62∼76	53∼72	WM	WM	14 d	⑤⑥⑦
Zhang, 2015	50	50	26/24	28/22	58.6 ± 9.2	60.3 ± 7.4	45∼72		WM	WM	14 d	①⑤⑥
Zou 2013	40	40	22/18	21/19	57.9 ± 9.8	58.7 ± 10.1	42∼75	41∼76	WM	WM	14 d	①⑦
Tian, 2020	81	81	52/29	50/31	63.5 ± 5.2	63.5 ± 5.1	51∼73	50∼72	WM	WM	NR	①③④⑤⑥

E, experiment group; C, control group; M, male; F, female; NR, not report; WM, western medicine; ①clinical effectiveness rate; ②the activities of daily living (ADL); ③low shear blood viscosity (LBV); ④high shear blood viscosity (HBV); ⑤plasma viscosity (PV); ⑥fibrinogen (FIB); ⑦adverse reactions (ADRs).

### 3.3 Quality evaluation

**FIGURE 3:**
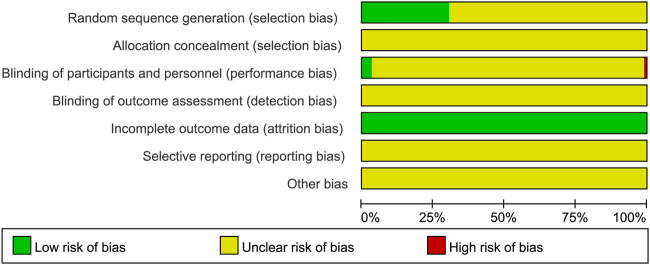
Risk of bias for the RCTs included in this NMA.

The risk of bias in RCTs included in this NMA was assessed using a tool developed by the Cochrane Collaboration (Higgins et al., 2011). In 35 RCTs, random sequences were generated using a random number table, considered as low risk in terms of selection bias; the allocation concealment was not clear. In terms of performance bias, four RCTs mentioned blinding, evaluated as low risk, and one RCT clearly pointed out that blinding was not used, considered to be high risk. In all RCTs, complete data were available, and the attrition bias was evaluated as low risk. Moreover, reporting bias and other bias were not clear. The risk of bias for the RCTs included in this NMA is described in Figure 3.

### 3.4 Outcomes

#### 3.4.1 Clinical effectiveness rates

**FIGURE 4:**
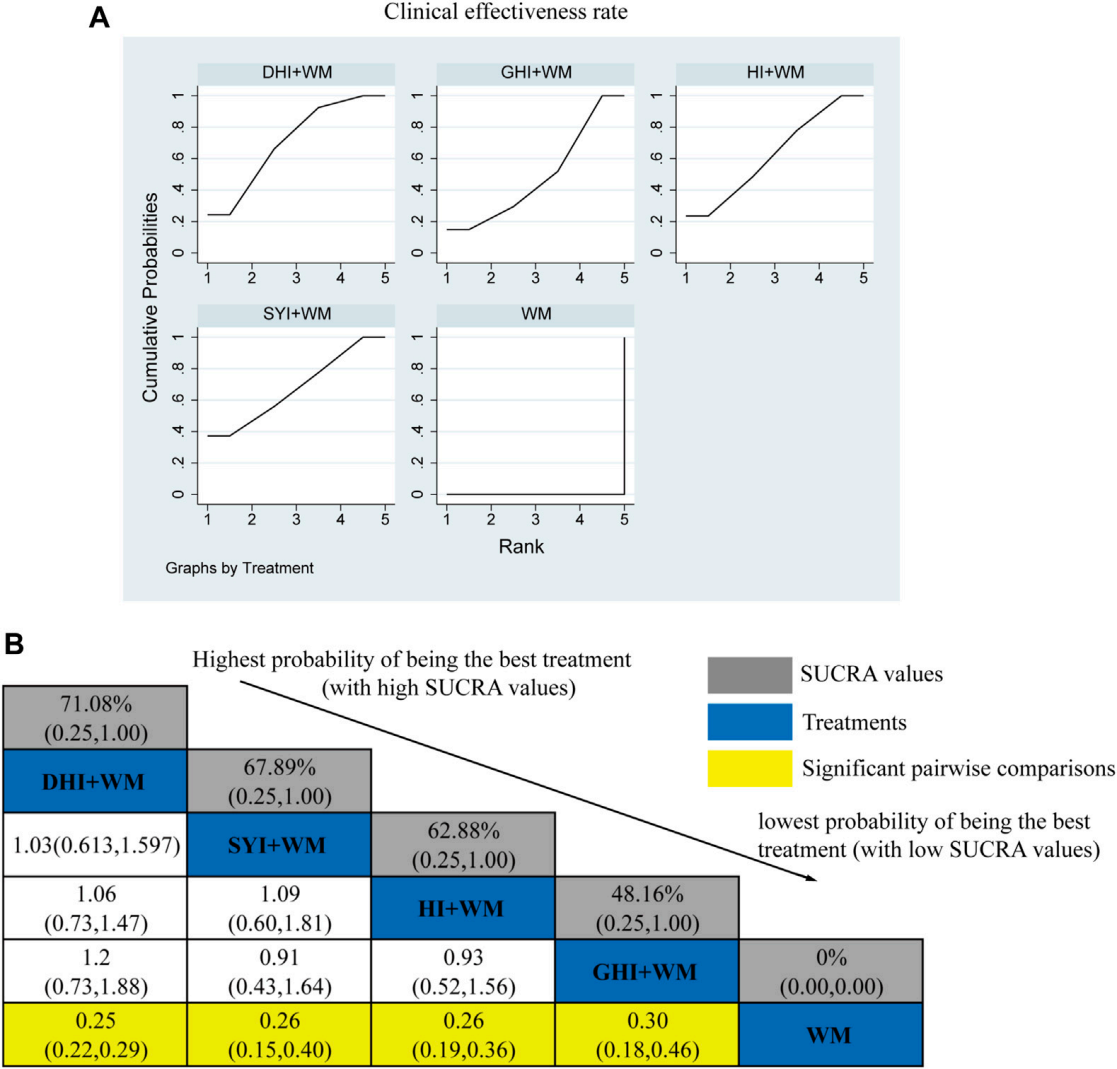
Relative effect sizes of the clinical effectiveness rate according to NWA. **(A)** SUCRA graph for the clinical effectiveness rate. **(B)** SUCRA values and ORs with 95% CIs of clinical effectiveness rates. Significant pairwise comparisons are highlighted in yellow.

A total of 104 RCTs compared the clinical effectiveness rates: HI + WM vs. WM (*n* = 14), SYI + WM vs. WM (*n* = 7), GHI + WM vs. WM (*n* = 8), and DHI + WM vs. WM (*n* = 75). As shown in Figure 4A, HCIs + WM treatments had significantly higher clinical effectiveness rates than WM alone: HI + WM (OR = 0.26, 95% CI: 0.19, 0.36), SYI + WM (OR = 0.26, 95% CI: 0.15, 0.40), GHI + WM (OR = 0.3, 95% CI: 0.18, 0.46), and DHI + WM (OR = 0.25, 95% CI: 0.22, 0.29). In addition, the results of a pairwise comparison of the four injections were as follows: HI + WM vs. SYI + WM (OR = 1.09, 95% CI: 0.60,1.81), HI + WM vs. GHI + WM (OR = 0.93, 95% CI: 0.52,1.56), HI + WM vs. DHI + WM (OR = 1.06, 95% CI: 0.73, 1.47), SYI + WM vs. GHI + WM (OR = 0.91, 95% CI: 0.43, 1.64), and GHI + WM vs. DHI + WM (OR = 1.2, 95% CI: 0.73, 1.88), indicating there were no significantly differences in the pairwise comparisons of the four types of HCI.

The SUCRA is shown in Figures 4A,B, DHI + WM (71.08%) >SYI + WM (67.89%) >HI + WM (62.88%) >GHI + WM (48.16%) >WM (0%). According to the SUCRA, DHI + WM treatment was the best intervention for improving the clinical effectiveness rate.

#### 3.4.2 ADL Function

**FIGURE 5:**
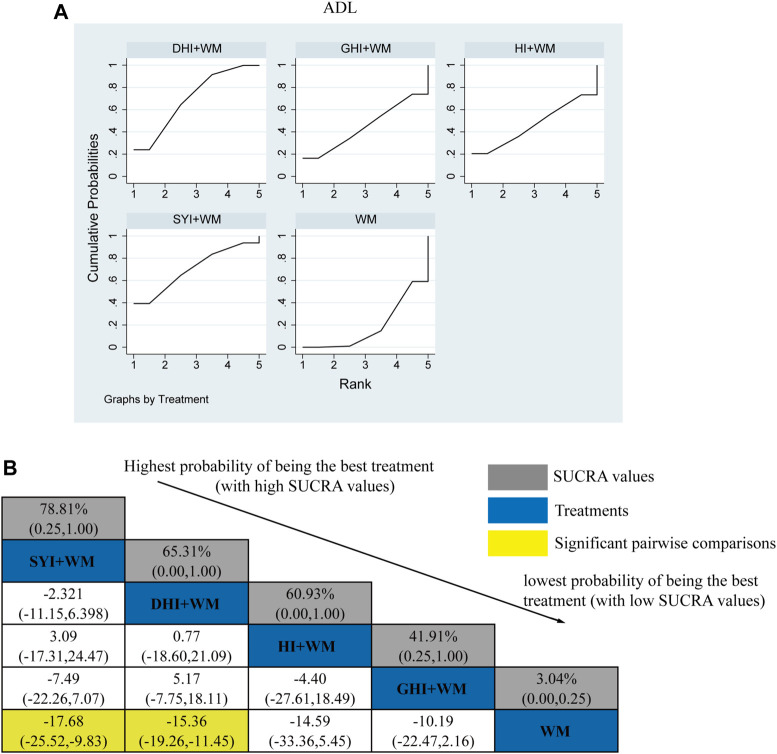
Relative effect sizes of the ADL according to NWA. **(A)** SUCRA graph for ADL. **(B)** SUCRA values and MDs with 95% CIs of the ADL. The significant pairwise comparisons are highlighted in yellow.

A total of 36 RCTs investigated ADL: HI + WM vs. WM (*n* = 2), SYI + WM vs. WM (*n* = 5), GHI + WM vs. WM (*n* = 4), and DHI + WM vs. WM (*n* = 23). Figure 5B shows the comparisons of SYI + WM vs. WM (MD = −17.68, 95% CI: −25.52,−9.83) and DHI + WM vs. WM (MD = −15.36, 95% CI: 19.26, −11.45). There were no significantly differences in the pairwise comparisons of other types of HCI.

The SUCRA is shown in Figures 5A,B, SYI + WM (78.81%), DHI + WM (65.31%), HI + WM (60.93%), and GHI + WM (41.91%), respectively. Therefore, SYI + WM treatment was probably the best effective intervention for improving theADL.

#### 3.4.3 LBV level

**FIGURE 6:**
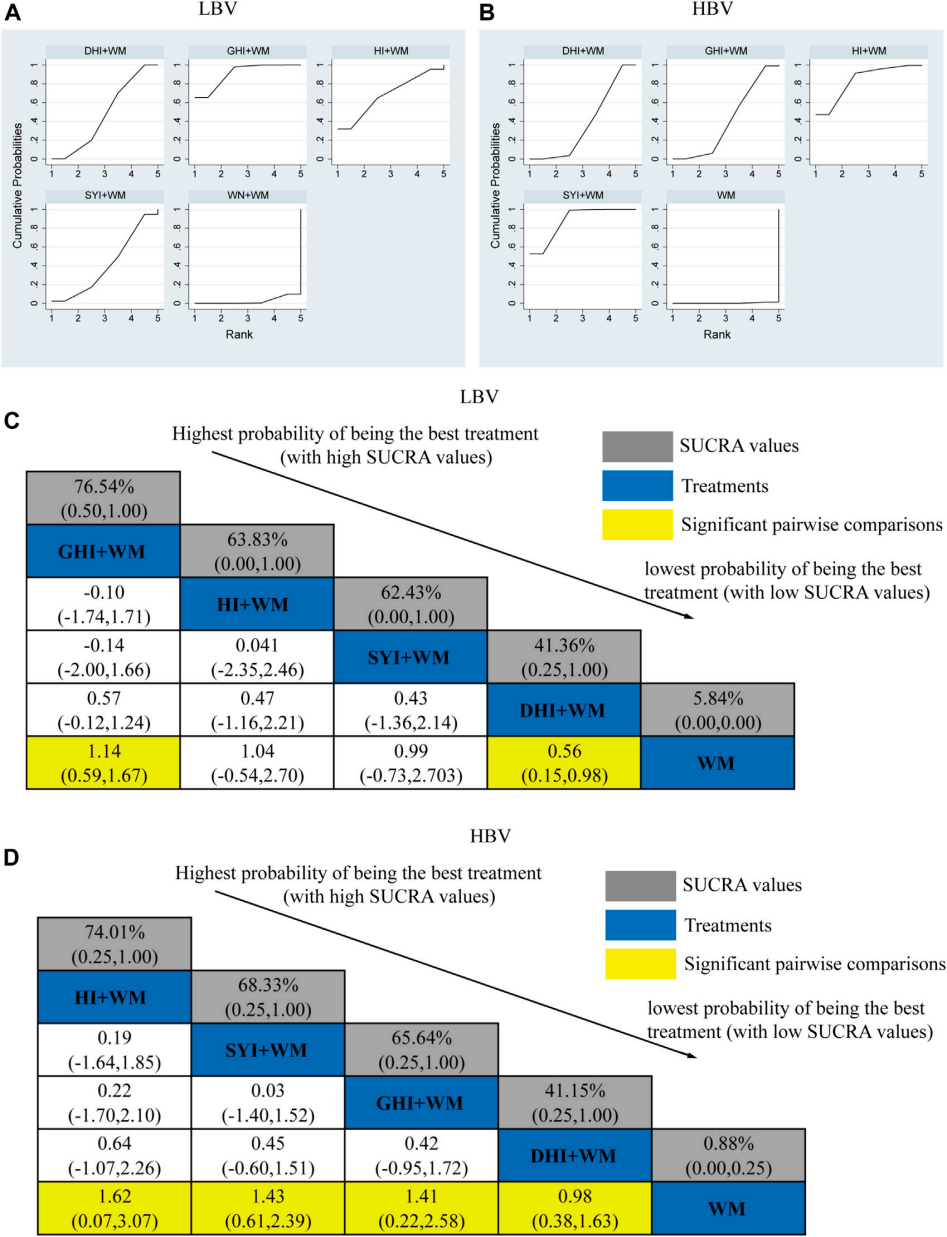
Relative effect sizes of the LBV and HBV according to NWA. **(A,B)** SUCRA graphs of the outcomes. **(C,D)** SUCRA values and MDs with 95% CIs. The significant pairwise comparisons are highlighted in yellow.

A total of 27 RCTs investigated the LBV: HI + WM vs. WM (*n* = 1), SYI + WM vs. WM (*n* = 3), GHI + WM vs. WM (*n* = 8), and DHI + WM vs. WM (*n* = 15). Figure 6C shows the comparisons of GHI + WM vs. WM (MD = 1.14, 95% CI: 0.59, 1.67) and DHI + WM vs. WM (MD = 0.56, 95% CI: 0.15, 0.98).

The SUCRA is shown in Figures 6A,C, GHI + WM (76.54%) > HI + WM (63.83%) > SYI + WM (62.43%) > DHI + WM (41.36%). According to the ranking of SUCRA, the GHI + WM (76.54%) treatment was probably to be the best intervention in neurological impairment.

#### 3.4.4 HBV level

A total of 26 RCTs investigated the HBV level: HI + WM vs. WM (*n* = 1), SYI + WM vs. WM (*n* = 3), GHI + WM vs. WM (*n* = 6), and DHI + WM vs. WM (*n* = 16). All CHIs combined with WM achieved a better effect in HBV than using WM alone. The significant results have been shown in Figure 6D, HI + WM vs. WM (MD = 1.62, 95% CI: 0.07, 3.07), SYI + WM vs. WM (MD = 1.43, 95% CI: 0.61, 2.39), GHI + WM vs. WM (MD = 1.41, 95% CI: 0.22, 2.58), and DHI + WM vs. WM (MD = 0.98, 95% CI: 0.38, 1.63).

The SUCRA is shown in Figures 6B,D, HI + WM (74.01%), SYI + WM (68.33%), GHI + WM (65.64%), and DHI + WM (41.15%), respectively. According to the SUCRA probabilities, the HI + WM treatment appeared to be the best interventions for HBV.

#### 3.4.5 PV level

**FIGURE 7:**
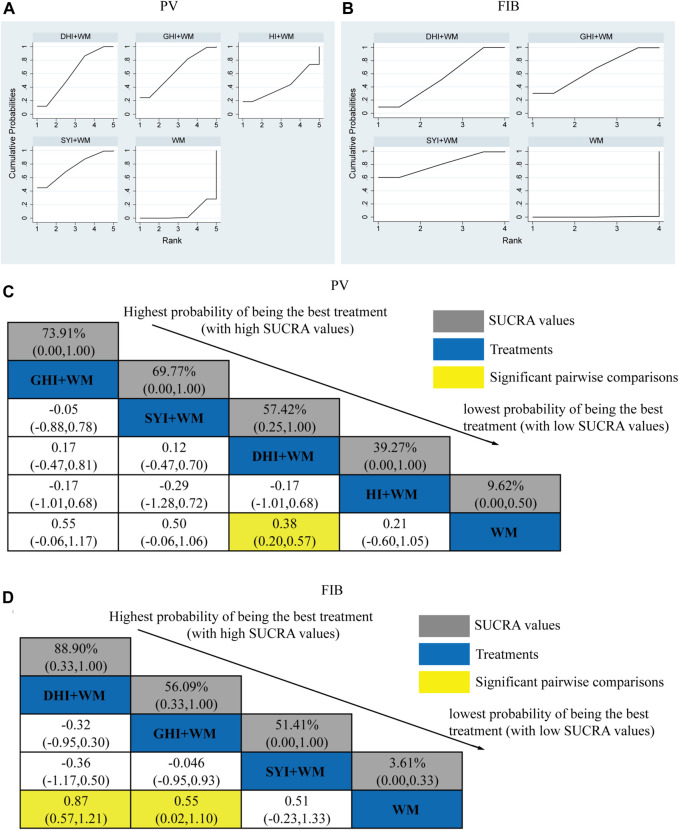
Relative effect sizes of PV and FIB according to NWA. **(A,B)** SUCRA graphs of the outcomes. **(C,D)** SUCRA values and MDs with 95% CIs. Significant pairwise comparisons are highlighted in yellow.

A total of 33 RCTs investigated the PV: HI + WM vs. WM (*n* = 1), SYI + WM vs. WM (*n* = 3), GHI + WM vs. WM (*n* = 4), and DHI + WM vs. WM (*n* = 25). The statistically significant results are shown in Figure 7C, DHI + WM vs. WM (MD = 0.38, 95% CI: 0.20, 0.57).

The SUCRA is shown in Figures 7A,C, GHI + WM (73.91%), SYI + WM (69.77%), DHI + WM (57.42%), and HI + WM (39.27%). GHI + WM treatment was probably to be the best intervention in plasma viscosity. However, GHI + WM vs. WM (MD = 0.55, 95% CI: −0.06, 1.17) and SYI + WM vs. WM (MD = 0.50, 95% CI: −0.06, 1.06), indicating there was no significant difference in the pairwise comparisons.

#### 3.4.6 FIB level

A total of 33 RCTs investigated the FIB level: HI + WM vs. WM (*n* = 0), SYI + WM vs. WM (*n* = 2), GHI + WM vs. WM (*n* = 4), and DHI + WM vs. WM (*n* = 27). The statistically significant results are shown in Figure 7D, DHI + WM vs. WM (MD = 0.87, 95% CIs: 0.57, 1.2), and GHI + WM vs. WM (MD = 0.55, 95% CIs: 0.02, 1.10).

The SUCRA is shown in Figures 7B,D, DHI + WM (88.90%), GHI + WM (56.09%), and SYI + WM (51.41%). Accordingly, DHI + WM treatment was the best intervention in decreasing the FIB level.

### 3.5 Cluster analysis

**FIGURE 8:**
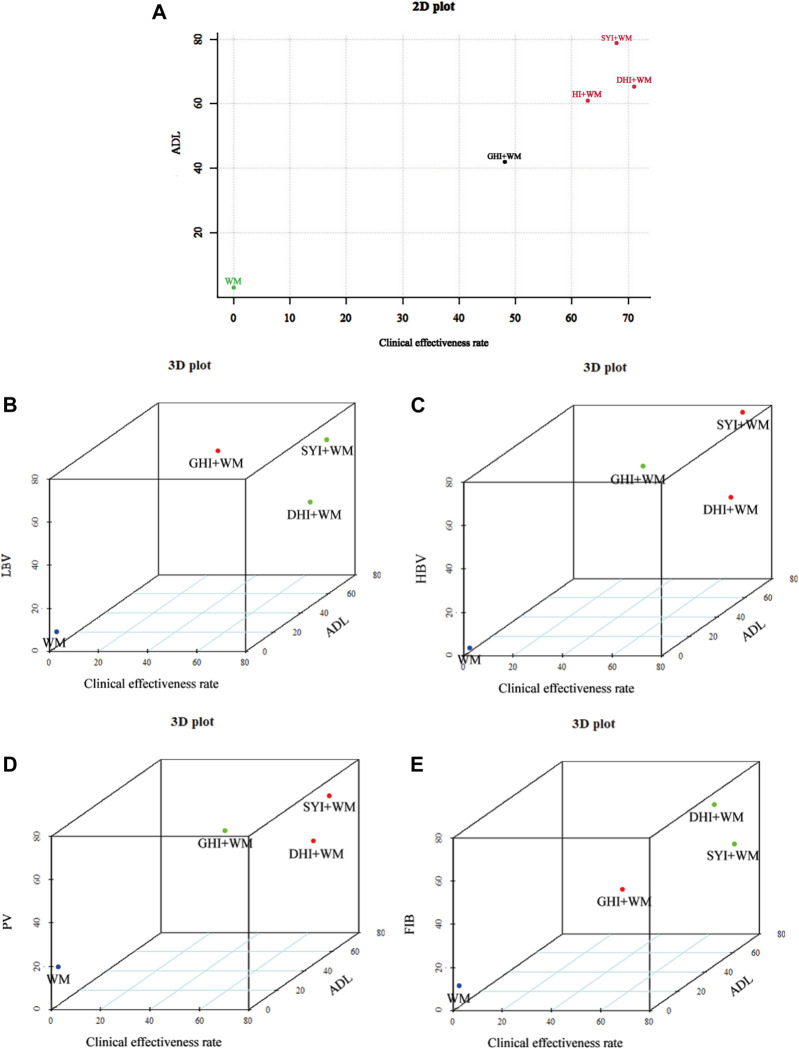
Cluster analysis plots. **(A)** Cluster analysis for clinical effectiveness rate (*X* axis) and ADL (*Y* axis). **(B)** Cluster analysis for clinical effectiveness rate (*X* axis), ADL (*Y* axis) and LBV (*Z* axis). **(C)** Cluster analysis for clinical effectiveness rate (*X* axis), ADL (*Y* axis) and HBV (*Z* axis). **(D)** Cluster analysis for clinical effectiveness rate (*X* axis), ADL (*Y* axis) and PV (*Z* axis). **(E)** Cluster analysis for clinical effectiveness rate (*X* axis), ADL (*Y* axis) and FIB (*Z* axis). Interventions with the same color belong to the same cluster.

When cluster analysis was performed to four HCIs that reported the clinical effectiveness rate and ADL, we found SYI + WM, DHI + WM and HI + WM were classified into the same category, indicating they exerted similar effectiveness (Figure 8A). In addition, we performed multidimensional cluster analysis on interventions with more than 1 included RCTs to further evaluate the comprehensive efficacy of HCIs. All the results demonstrated DHI + WM and SYI + WM might have better therapeutic effects (Figures 8B–E).

### 3.6 Safety evaluation

Of the 120 RCTs, 59 trials investigated the ADRs/ADEs, involving a total of 5,749 patients. Overall, 2,778 cases in the control group (WM), and 2,971 cases in the experiment group (HCIs + WM) were included.

In terms of the safety evaluation of HI, six RCTs were included, involving 674 patients, 320 cases in the WM group and 354 cases in the HI + WM group. There were three cases of ADRs that occurred in the HI + WM group: pruritus (2 cases) and skin flushing (1 case).

In terms of the safety evaluation of SYI, five RCTs were included, involving 554 patients, 277 cases in the WM group and 277 cases in the SYI + WM group. There were seven cases of ADRs that occurred in the SYI + WM group: rash (2 cases), gastrointestinal reactions (4 cases), such as nausea, vomiting and diarrhea, and gingival bleeding (1 case). Five cases of ADRs occurred in the WM group: gastrointestinal reactions (3 cases) and gingival bleeding (2 cases).

In terms of the safety evaluation of GHI, seven RCTs were included, involving 1055 patients, 514 cases in the WM group, and 541 cases in the GHI + WM group. There were 23 cases of ADRs in the GHI + WM group: 10 cases of gastrointestinal reactions, such as nausea, vomiting, and diarrhea, cases of headache or dizziness (5 cases), rash (3 cases), vasculitis (1 case), lethargy (2 cases), and mild fever (2 cases). 29 cases of ADRs occurred in the WM group: gastrointestinal reactions (11 cases), headache or dizziness (8 cases), rash (1 case), gingival bleeding (2 cases), vasculitis (2 cases), 5 cases of lethargy, and mild abnormal liver function (2 cases).

In terms of the safety evaluation of DHI, 41 RCTs were included, involving 3,847 patients, 1,848 cases in the WM group, and 1,999 cases in the DHI + WM group. There were 53 cases of ADRs that had occurred in the DHI + WM group: gastrointestinal reactions (18 cases), headache or dizziness (19 cases), rash (7 cases), vasculitis (1 case), fatigue (1 case), 1 case of hypotension, mild palpitation (2 cases), limb pain (2 cases), and cough (2 cases). 64 cases of ADRs occurred in the WM group: gastrointestinal reactions (17 cases), headache or dizziness (19 cases), rash (9 cases), vasculitis (2 cases), fatigue (1 case), hypotension (3 cases), mild palpitation (3 cases), epigastric discomfort (4 cases), limb pain (3 cases), and cough (3 cases).

### 3.7 Publication bias

**FIGURE 9:**
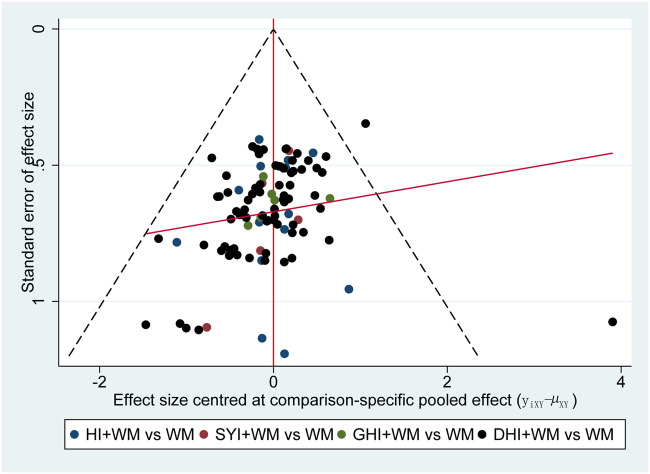
Funnel plot of clinical effectiveness rate.

The funnel plot of the clinical effectiveness rate was not quite symmetrical, indicating the potential publication bias in this NMA (Figure 9).

### 3.8 Consistency test

In this NMA, as there were no closed loops, an overall consistency test was not possible. All the evidences about these contrasts were obtained from the trials, which directly compare them. The local consistency test showed that *p* > 0.05, indicating that there was no local inconsistency in this study.

## 4 Discussion

We performed a NMA on four common HCIs and compared the outcomes to determine the most appropriate choice, providing some references for the clinical treatment of AIS. This NMA included 120 RCTs involving 12,658 patients, evaluating the clinical effectiveness rate, ADL, LBV HBV, PV, FIB levels, and ADRs after the application of four HCIs combined with WM and WM alone. The clinical effectiveness rate, evaluated by neurological function recovery, was considered as the primary outcome. According to the ranking of SUCRA, DHI + WM treatment was the best intervention for improving the clinical effectiveness rate, while SYI + WM was the best treatment for ADL. Moreover, multidimensional cluster analysis demonstrated that DHI + WM and SYI + WM might have a better comprehensive therapeutic effect.

DHI is extracted from Danshen (*Salvia miltiorrhiza Bunge, Lamiaceae, Salviae miltiorrhizae radix et rhizoma*) and Honghua, widely used in the treatment of AIS. The main active components of DHI are danshensu, protocatechuic aldehyde, safflower yellow A, and salvianolic acid (Jiang et al., 2015; Liu et al., 2019). The clinical trial has shown that the value of DHI in treating AIS with blood stasis syndrome is comprehensively evaluated as Grade A (Cui et al., 2021). Moreover, pharmacological studies have shown that DHI can protect the blood-brain barrier after AIS (Zeng et al., 2021a), improve the energy metabolism of cells in the ischemic area (Zeng et al., 2021b), enhance the mitochondrial function (Orgah et al., 2019), exert anti-inflammatory, anti-apoptotic, and antioxidant effects (Wang et al., 2020a), thereby improving neurological impairment. SYI is extracted from Honghua, its main component is safflower yellow. A clinical trial on IS has shown that SYI may treat AIS by alleviating the inflammation reaction of patients (Li et al., 2015a). The main active component of safflower yellow is hydroxysafflower yellow A (HYSA), with antioxidant activity. Pharmacological studies have demonstrated that HYSA plays an important role in anti-atherosclerosis (Xue et al., 2021), protecting neurons from excitotoxic damage (Wang et al., 2016), dilating cerebral vessels, and improving the cerebrovascular permeability (Sun et al., 2018; Li et al., 2022).

Atherosclerosis is the pathological basis of IS. Abnormal hemorheological indexes, especially the increase of plasma viscosity and fibrinogen levels, will accelerate the pathological process of atherosclerosis and lead to stroke. Furthermore, a previous study has shown that hemorheological disturbances affect cognitive functions of IS patients (Velcheva and Nikolova, 2008). Ameliorating hemorheology is of great significance to the prognosis of stroke (Resch, 1992). In this NMA, we found that GHI had the best effect in reducing PV and LBV and DHI had the best effect in reducing FIB. As there was only one RCT in HI treatment, it could not prove that the curative effect of HI was better than other HCIs. GHI comprised safflower aqueous extract and aceglutamide. Several studies have shown that the mechanisms of GHI in treating IS were related to inhibiting inflammation and reducing neuronal apoptosis (Wang et al., 2022; Zhang et al., 2022). During inflammation, the hemorheological system is impaired (Pretorius, 2018). The regulation of hemodynamics by GHI may be related to its anti-inflammatory effects.

In addition to the clinical efficacy, the safety of CHIs in treating AIS is also particularly important. In this NMA, 59 RCTs investigated the ADRs/ADEs (HI 6 RCTs; SYI five RCTs; GHI seven RCTs and DHI 41 RCTs), more than 50% of RCTs did not report safety. However, more than 50% of RCTs did not report them. Therefore, we were unable to make the accurate conclusions of HCIs’ safety. Previous studies had shown that most ADRs of DHI were mild and moderate. Post-marketing safety monitoring and re-evaluation studies of DHI with 30,888 cases show that the incidence of ADRs/ADEs was 3.50‰ (Li et al., 2015b). Unfortunately, there is a lack of large samples and high-quality safety research on other HCIs post-marketing. Since there are few studies focused on safety assessments in this NMA, further experimental and clinical evidence is required to verify the safety of these HCIs. Moreover, in order to improve the safety of clinical applications of TCMIs, a research system and post-marketing safety surveillance and re-evaluation should be established.

This study is a NMA based on RCTs, using a Bayesian algorithm, which has some advantages. A comprehensive multi-platform literature search was conducted for this study and strict inclusion and exclusion criteria were formulated. Importantly, this is the first NMA to evaluate the clinical efficacy of HCIs + WM in treating AIS. We also analyzed the changes of hemorheological indexes and the safety of HCIs, which has certain significance for guiding the treatment of AIS.

However, this study still has some limitations. First, the current NMA-included RCTs were observed to have a selection bias and performance bias; there was a lack of information regarding allocation concealment and blinding of participants or personnel in most RCTs. Second, apart from DHI + WM, there were few eligible RCTs for other interventions, which might lead to bias of the outcomes. Third, most of the included RCTs were single-center studies, and the patients included in the studies may be regionalized. Additionally, all of the RCTs in this NMA were conducted among Chinese population. Whether the curative effect is affected by race or region is still unclear. Importantly, clinical heterogeneity might happen due to the diversity of conventional WM treatment and the different time of onset, dosage and the course of treatment. Moreover, few eligible RCTs reported follow-up results; the recurrence and mortality rates of patients treated with HCI + WM are not well understood. In view of the above limitations, RCTs should be conducted more standardized, clearly defined in terms of populations, interventions, comparators, outcomes, and study designs (PICOS). In order to accurately evaluate the efficacy and safety of TCMIs and promote their application in the real-world clinical practice, the methodological quality of RCTs should be improved.

## 5 Conclusion

In conclusion, based on the results of this Bayesian NMA, HCIs combined with WM treatments significantly improve the therapeutic effect, and DHI and SYI combined with WM treatments are probably preferred among HCIs for the treatment of AIS. However, due to the limitations, this conclusion may be biased. Importantly, high-quality, multicenter, and double-blind RCTs should be performed in the future to validate our findings. Additionally, we need to improve the quality of TCMI security assessment in RCTs, strictly monitor the ADRs/ADEs of TCMI, and standardize medication.

## Data Availability

The original contributions presented in the study are included in the article/Supplementary Material, further inquiries can be directed to the corresponding authors.
