# Supplementary material for: Comparative efficacy of Honghua class injections for treating acute ischemic stroke: A Bayesian network meta-analysis of randomized controlled trials
Source: Front Pharmacol. 2022 Sep 28;13:1010533. doi: 10.3389/fphar.2022.1010533 (PMC9554475; doi:10.3389/fphar.2022.1010533)
Supplement: Supplementary file 1 [file DataSheet1.zip › Supplementary file/Supplementary Material S4.doc]

Supplementary Material S4**:** The characteristic details of the RCTs and the included literatures.

**Table 1** Characteristics details of the studies included in this NMA.

| **study ID** | **Sample size** | | **Sex(M/F)** | | **Average age** | | **Age range** | | **Therapy of  experiment group** | **Therapy of  control group** | **Course** | **outcomes** |
| --- | --- | --- | --- | --- | --- | --- | --- | --- | --- | --- | --- | --- |
|  | **C** | **E** | **C** | **E** | **C** | **E** | **C** | **E** |  |  |  |  |
| Jia,2021 | 49 | 50 | 26/23 | 25/25 | 62.15±8.26 | 61.86±8.06 | 50~78 | 48~78 | HI 20mL+WM | WM | 14 d | ② |
| Wang, 2018 | 30 | 30 | NR | | NR | | NR | | HI 20mL+WM | WM | 14 d | ① |
| Li, 2016 | 116 | 117 | 69/47 | 67/50 | 65.37±1.24 | 64.35±1.02 | 38~80 | 37~79 | HI 18mL+WM | WM | NR | ①⑦ |
| Zhang,2013 | 25 | 25 | 13/12 | 12/13 | 74.6±7.1 | 76.3±6.7 | 65~82 | 68~83 | HI 40mL+WM | WM | 28 d | ①⑦ |
| Liu,2013 | 30 | 30 | 15/15 | 14/16 | 63±13 | 64±13 | NR | | HI 20mL+WM | WM | 14 d | ① |
| Li.,2012 | 60 | 60 | 81/39 | | 48.32±10.56 | | 32~63 | | HI 40mL+WM | WM | 10 d | ① |
| Liu,2012 | 70 | 66 | 38/32 | 36/30 | 61.5 | 63.4 | 38~80 | 43~79 | HI 40mL+WM | WM | 14 d | ① |
| Zhao,2011 | 68 | 68 | 39/29 | 37/31 | 62.1 | 61.8 | 43~82 | 41~83 | HI 40mL+WM | WM | 14 d | ① |
| Ge,2011 | 44 | 62 | 37/25 | 27/17 | NR | | 48~78 | 52~83 | HI 15mL+WM | WM | 20 d | ①⑦ |
| Lian 2010 | 100 | 100 | 80/20 | 78/22 | 58.1 | 57.6 | 44~67 | 45~71 | HI 20mL+WM | WM | 28 d | ① |
| Li,2010 | 28 | 28 | 15/13 | 18/10 | 51.7±10.9 | 52.3±11.1 | 41~69 | | HI 15mL+WM | WM | 15-20 d | ① |
| Xu 2005 | 50 | 50 | 32/18 | 28/22 | 64±10 | 59±10 | 51~68 | 49~75 | HI 20mL+WM | WM | 14 d | ①⑦ |
| Lv,2005 | 74 | 76 | 39/35 | 40/36 | 65.9±8.1 | 66.4±8.3 | 51~70 | 50~73 | HI 15mL+WM | WM | 15 d | ① |
| Liu 2004 | 55 | 55 | 31/24 | 29/26 | NR | | 49~75 | 48~73 | HI 20mL+WM | WM | 21 d | ①③④⑤⑦ |
| Song,2002 | 30 | 45 | 16/14 | 27/18 | 59.64 | 60.5 | 38~73 | 40~81 | HI 20mL+WM | WM | 14 d | ①②⑦ |
| Song, 2021 | 68 | 68 | 73/63 | | 64.7±10.01 | | 49~82 | | SYI 100mg+WM | WM | 14 d | ①②⑦ |
| Jiang, 2022 | 82 | 82 | 50/32 | 45/37 | 71.18± 3.29 | 71.12± 3.22 | NR | | SYI 100mg+WM | WM | 14 d | ①②③④⑥ |
| Tang 2013 | 46 | 46 | 23/23 | 24/22 | 51.6±10.4 | 52.1±9.5 | 43~73 | 45~72 | SYI 100mg+WM | WM | 14 d | ①⑦ |
| Cheng 2019 | 60 | 60 | 40/20 | 41/19 | 63.23±5.23 | 63.26±5.21 | 44~79 | 45~78 | SYI 100mg+WM | WM | 14 d | ①②③④⑤⑦ |
| Guo 2016 | 40 | 40 | 25/15 | 26/14 | 63.5±15.2 | 61.4±13.6 | 45~82 | 42~80 | SYI 100mg+WM | WM | 14 d | ①②⑦ |
| Ji,2016 | 48 | 48 | 26/22 | 25/23 | 63.32±5.51 | 63.26±5.47 | 50~72 | 51~73 | SYI 150mg+WM | WM | 14 d | ①②⑤ |
| Huang,2011 | 63 | 63 | 42/21 | 44/19 | 53.2 | 52.8 | 42~82 | 43~80 | SYI 100mg+WM | WM1 | 14 d | ①⑦ |
| Li 2015 | 31 | 66 | 9/21 | 28/38 | 58.84 ± 10.18 | 59.47 ± 10.92 | 30~70 | 31~70 | SYI 80mg+WM | WM | 14 d | ③④⑤⑥ |
| Chen 2022 | 42 | 42 | 22/20 | 24/18 | 62.53±1.24 | 62.63±2.13 | 46～78 | 46～80 | GHI 15mL+WM | WM | 14 d | ② |
| Song, 2021 | 40 | 40 | 17/13 | 26/14 | 63.37±5.58 | 63.72±6.03 | 49～71 | 50~72 | GHI 20mL+WM | WM | 14 d | ③④⑤⑥ |
| Zhao 2021 | 77 | 77 | 37/40 | 43/34 | 59.37±4.56 | 61.13±4.90 | 40～80 | 41~78 | GHI 20mL+WM | WM | 14 d | ①③④⑤⑥⑦ |
| Lu 2022 | 52 | 52 | 28/24 | 29/23 | 57.45±5.98 | 57.33±5.90 | 37~75 | 37~75 | GHI 20mL+WM | WM | 14 d | ①②⑤⑦ |
| Xiao,2020 | 45 | 45 | 24/21 | 25/20 | 68.14±2.32 | 69.15.±2.44 | 58~75 | 60~75 | GHI 20mL+WM | WM | 14 d | ①②⑦ |
| Jiang,2016 | 40 | 40 | 20/20 | 19/21 | NR | | 41~75 | 40~75 | GHI 20mL+WM | WM | 14 d | ③④⑤ |
| Sheng,2019 | 38 | 38 | 23/15 | 21/17 | 63.24±9.48 | 64.08±9.16 | 45~82 | 46~82 | GHI 20mL+WM | WM | 14 d | ①③④⑦ |
| Li,2018 | 30 | 30 | 18/12 | 18/12 | 63.1±5.2 | 63.5±5.3 | 46~80 | 46~80 | GHI 20mL+WM | WM | 10-15 d | ①③④⑦ |
| Li,2018 | 68 | 68 | 41/27 | 39/29 | 61.9±7.2 | | 46~83 | 48~85 | GHI 20mL+WM | WM | 14 d | ①②③④ |
| Hu 2009 | 52 | 60 | 27/25 | 32/28 | 60.5±7.3 | 60.8±7.2 | 47~76 | 45~78 | GHI 20mL+WM | WM | 14 d | ①③⑥⑦ |
| Tang 2016 | 39 | 39 | 21/18 | 20/19 | 62.75±5.58 | 62.35±5.53 | 53~75 | 52~71 | GHI 20mL+WM | WM | 14 d | ①③⑥ |
| Zhang,2010 | 220 | 239 | 143/77 | 138/101 | 61.17±11.68 | 62.22±10.22 | NR | | GHI 15mL+WM | WM | 21 d | ⑦ |
| Wu, 2022 | 30 | 31 | 18/12 | 21/10 | 50.27±4.63 | 50.25±4.78 | 45~75 | | DHI 20mL+WM | WM | 14 d | ①⑤⑦ |
| He, 2021 | 30 | 30 | 19/11 | 20/10 | 68.61±7.96 | 67.09±8.57 | 54~80 | 55~82 | DHI 20mL+WM | WM | 14 d | ①③④⑥ |
| Wang 2021 | 68 | 68 | 46/22 | 44/24 | 60.15±5.33 | 61.28±5.29 | 48~94 | | DHI 20mL+WM | WM | 14 d | ①② |
| Shen,2021 | 50 | 50 | 23/27 | 22/28 | 65.42±5.87 | 65.39±5.59 | 53~83 | 52~82 | DHI 30mL+WM | WM | 14 d | ①② |
| Zhang 2021 | 43 | 43 | 24/19 | 22/21 | 72.17±3.42 | 72.43±3.58 | 60~88 | 60~88 | DHI 20mL+WM | WM | 14 d | ②⑦ |
| Wang 2021 | 45 | 45 | 33/12 | 31/14 | 62.8±4.5 | 62.3±4.6 | 41~70 | 42~70 | DHI 20mL+WM | WM | 14 d | ①②⑥ |
| Yuan 2018 | 40 | 40 | 24/16 | 23/17 | 51. 6±2. 5 | 52.3±3.2 | 25~80 | 24~80 | DHI 20mL+WM | WM | 14 d | ①② |
| Yuan,2019 | 38 | 38 | 20/18 | 21/17 | 65.39±2.19 | 65.50±2.31 | 51~73 | 52~74 | DHI 20mL+WM | WM | 14 d | ①②⑥⑦ |
| Fan 2019 | 68 | 68 | 41/27 | 37/31 | 58. 31±10. 05 | 60. 04±10. 53 | 44~76 | 46~78 | DHI 20mL+WM | WM | 21 d | ①②⑦ |
| Wu,2019 | 42 | 42 | 27/15 | 25/17 | 66.1±7.3 | 66.8±7.1 | 48~82 | 49~84 | DHI 20mL+WM | WM | 14 d | ①②③④⑤⑥ |
| Li 2020 | 52 | 52 | 26/26 | 29/23 | 68.58±4.68 | 68.34±5.64 | 60~79 | 60~79 | DHI 30mL+WM | WM | 14 d | ①⑤⑥⑦ |
| Tang 2019 | 45 | 41 | 23/22 | 21/20 | 46.5±7.9 | 48.7±8.3 | 37~73 | 38~76 | DHI 20mL+WM | WM | 14 d | ①②⑦ |
| Zhang 2018 | 54 | 54 | 29/25 | 28/26 | 60.2±3.1 | 61.8±3.2 | 49~72 | 51~74 | DHI 30mL+WM | WM | 14 d | ①⑤⑥ |
| Kang et al.,2020 | 60 | 65 | 35/25 | 37/28 | 54. 95±5. 93 | 55. 42±5. 99 | 41~79 | 40~81 | DH 20mL+WM | WM | 14 d | ② |
| Chen,2019 | 165 | 165 | 86/79 | 83/82 | 59.8±6.7 | 60.1±6.2 | 36~72 | 37~73 | DHI 20mL+WM | WM | 15 d | ①②⑥ |
| Liu,2019 | 49 | 49 | 24/25 | 25/24 | 59.32±12.14 | 58.91±12.23 | NR | | DHI 30mL+WM | WM |  | ①③④⑤⑥ |
| Liu 2020 | 71 | 71 | 41/30 | 43/28 | 61.97±8.32 | 63.24±6.57 | 43~75 | 47~78 | DHI 20mL+WM | WM | 14 d | ①② |
| Zhu 2020 | 30 | 30 | 16/14 | 18/12 | 57.62 ±4.87 | 56.34 ±5.29 | NR | | DHI 20mL+WM | WM | 14 d | ①② |
| Dai,2018 | 47 | 47 | 22/25 | 26/21 | 56.43±2.42 | 56.42±2.43 | 36~79 | 36~78 | DHI 20mL+WM | WM | 15 d | ①②⑦ |
| Qiao,2010 | 30 | 30 | 20/10 | 19/11 | NR | | 43~80 | 44~79 | DHI 20mL+WM | WM | 14 d | ① |
| Zhou,2009 | 50 | 50 | 35/15 | 36/14 | NR | | 44~77 | 45~78 | DHI 20mL+WM | WM | 14 d | ① |
| Gu 2012 | 80 | 80 | 41/39 | 38/42 | 59.24 | 60.31 | NR | | DHI 30mL+WM | WM | 15 d | ①③④⑤⑥⑦ |
| Zhang,2010 | 60 | 60 | 40/20 | 38/22 | 69.3 | 68.9 | 42~80 | 43~79 | DHI 40mg+WM | WM | 14 d | ①⑦ |
| Wang 2017 | 50 | 50 | 52/48 | | 53.6 | | 41~78 | | DHI 60mL+WM | WM | 14 d | ①⑦ |
| Tan 2016 | 43 | 43 | 49/37 | | NR | | 39~82 | | DHI 30mL+WM | WM | 14 d | ①⑦ |
| Liu 2010 | 40 | 40 | 23/17 | 22/18 | NR | | 51~57 | 52~70 | DHI 40mL+WM | WM | 15 d | ① |
| Yi 2010 | 40 | 40 | 28/12 | 24/16 | 57±7.5 | 55±6.5 | 44~78 | 45~76 | DHI 40mL+WM | WM | 14 d | ⑤⑦ |
| Pang 2011 | 80 | 85 | NR | | NR | | 45~78 | | DHI 40mL+WM | WM | 14 d | ①⑦ |
| Jiang 2008 | 40 | 40 | 24/16 | 23/17 | 59.50±11.75 | 59.82±9.45 | 35~85 | 36~86 | DHI 30mL+WM | WM | 14 d | ①⑦ |
| Wang,2008 | 30 | 30 | 14/16 | 17/13 | 64.1 | 63.7 | 49~83 | 50~85 | DHI 20mL+WM | WM | 15 d | ③④⑤⑦ |
| Hu 2008 | 32 | 32 | 21/11 | 18/14 | 59.1±9.7 | 62.1±5.7 | 56~75 | | DHI 40mL+WM | WM | 14 d | ③④⑤⑥ |
| Zheng 2015 | 84 | 84 | 51/33 | 53/31 | 50.21±8.66 | 49.67±8.27 | 33~69 | 36~68 | DHI 20-40mL+WM | WM | 14 d | ①⑤⑥ |
| Yun 2017 | 31 | 31 | 20/11 | 17/14 | 68.2±7.1 | 69.5±7.3 | 58~80 | | DHI 30mL+WM | WM | 14 d | ③④⑤⑥⑦ |
| Wang,2013 | 36 | 34 | 22/14 | 21/13 | 56.6±7.4 | 56.3±7.2 | 36~84 | 34~85 | DHI 20mL+WM | WM | 14 d | ①⑦ |
| An 2015 | 35 | 35 | 22/13 | 20/15 | 57±15 | 59±15 | 43~78 | 45~80 | DHI 30mL+WM | WM | 14 d | ① |
| Li,2017 | 30 | 30 | 18/12 | 19/11 | 62.03 ±4.11 | 61.48 ±3.95 | 42~70 | 41~71 | DHI 40mL+WM | WM | 14 d | ①⑥⑦ |
| Zhang 2016 | 40 | 40 | 48/32 | | 67.5 | | NR | | DHI 30mL+WM | WM | 14 d | ①③④⑤⑥⑦ |
| Yan,2013 | 59 | 57 | 32/27 | 31/26 | NR | | 41~73 | 43~72 | DHI 30mL+WM | WM | 14 d | ①⑦ |
| Gao,2011 | 30 | 32 | 16/14 | 17/15 | 65.5 | 63.2 | 45~76 | 40~79 | DHI 30mL+WM | WM | 14 d | ① |
| Zhang,2012 | 57 | 64 | 38/19 | 43/21 | 69.7±12.8 | 71.4±11.3 | 51~77 | 52~79 | DHI 30mL+WM | WM | 14 d | ① |
| Huang,2017 | 57 | 63 | 72/68 | | 64.37±1.56 | 64.61±2.34 | NR | | DHI +WM | WM | 28 d | ①⑦ |
| Li,2014 | 32 | 32 | 20/12 | 22/10 | 52±9.8 | 54±10.1 | 43~68 | 45~70 | DHI 20mL+WM | WM | 14 d | ①⑦ |
| Wu 2011 | 40 | 40 | 18/14 | 20/12 | 62±7 | 62±5 | NR | | DHI 20mL+WM | WM | 28 d | ① |
| Ma,2017 | 42 | 40 | 27/15 | 24/16 | 65.26±7.23 | 60.01±7.86 | 49~75 | 39~70 | DHI 20mL+WM | WM | 14 d | ①⑥⑦ |
| Guan 2017 | 40 | 40 | 26/14 | 25/15 | 58.79±5.78 | 59.83±5.16 | 41~75 | 43~74 | DHI 30mL+WM | WM | 14 d | ①③④⑤⑦ |
| Ma 2012 | 50 | 50 | 29/21 | 28/22 | 62.2±8.1 | 61.3±7.9 | 42~81 | 41~79 | DHI 20mL+WM | WM | 14 d | ① |
| Xiao,2016 | 35 | 35 | 21/14 | 23/12 | 59.0±9.7 | 59.0±9.7 | NR | | DHI 30mL+WM | WM | 30 d | ①②③④⑤⑥⑦ |
| Yang 2017 | 42 | 42 | 27/15 | 26/16 | 52.2±5.2 | 52.9±5.1 | 46~72 | 47~72 | DHI 40mL+WM | WM | 14 d | ①⑥ |
| Zhang,2017 | 45 | 45 | 26/19 | 24/21 | 69.3±9.6 | 68.9±9.5 | 60~85 | 59~84 | DHI 20mL+WM | WM | 14 d | ①⑤ |
| Zeng 2016 | 55 | 55 | 31/24 | 30/25 | 63.3±5.3 | 63.0±5.2 | 38~74 | 38~74 | DHI 20mL+WM | WM | 15 d | ①② |
| Mao,2010 | 29 | 29 | 18/11 | 17/12 | 61.1 | 61.3 | 43~73 | 45~75 | DHI 20mL+WM | WM | 14 d | ① |
| Zhang,2015 | 90 | 90 | 88/92 | | 51.2±5.4 | | 45~67 | | DHI 140mg+WM | WM | 14 d | ①⑦ |
| Li,2011 | 32 | 32 | 19/13 | 22/10 | 67.3 | 65.8 | 43~79 | 42~77 | DHI 20mL+WM | WM | 14 d | ⑦ |
| Shi,2010 | 40 | 43 | 28/12 | 29/14 | 64 | 65 | 41~78 | 42~79 | DHI 20mL+WM | WM | 14 d | ①⑦ |
| Zhao 2010 | 96 | 96 | 53/43 | 59/37 | 65.60±3.44 | 67.40±3.48 | 53~79 | 52~80 | DHI 20mL+WM | WM | 14 d | ①②④⑥ |
| Cai 2009 | 100 | 120 | 64/36 | 76/44 | 59.8 | 58.7 | 40~74 | 41~72 | DHI 20mL+WM | WM | 30 d | ①③④⑤⑥ |
| Wang 2012 | 40 | 80 | 28/12 | 52/28 | 63.6±11.2 | 64.7±10.5 | 43~83 | 45~81 | DHI 20mL+WM | WM | 20 d | ①⑦ |
| Zhang 2011 | 60 | 60 | 36/24 | 38/22 | 63.4±13.18 | 62.4±11.06 | 42~83 | 40~81 | DHI 20mL+WM | WM |  | ①③④⑤⑥ |
| Chen,2008 | 54 | 52 | 36/18 | 38/14 | 65.7±7.39 | 63.0±6.7 | 51~75 | 50~72 | DHI 40mL+WM | WM | 14 d | ①⑦ |
| Huang,2010 | 76 | 76 | 47/29 | 49/27 | 62.0±2.5 | 63.5±3.5 | NR | | DHI 20mL+WM | WM | 14 d | ①⑤⑥ |
| Zhou,2007 | 30 | 30 | 20/10 | 19/11 | NR | | 43~80 | 44~79 | DHI 20mL+WM | WM | 14 d | ① |
| Fan 2018 | 35 | 35 | NR | | 50.45±9.56 | 49.32 ±9.37 | 32~78 | 34~76 | DHI 40mL+WM | WM | 14 d | ①② |
| Lv,2017 | 42 | 42 | 28/14 | 25/17 | 58. 8 ± 8. 7 | 59. 2 ±8. 6 | 42~79 | 40~78 | DHI 40mL+WM | WM | 14 d | ①② |
| Liu,2015 | 40 | 40 | 45/35 | | 66.3±1.4 | | 50~88 | | DHI 20mL+WM | WM | 14 d | ① |
| Zhang 2012 | 60 | 60 | 31/29 | 32/28 | 64.0±12.3 | 63.3±11.5 | 28~83 | 32~81 | DHI 30mL+WM | WM | 14 d | ② |
| Yue,2012 | 30 | 30 | 18/12 | 19/11 | NR | | 40~77 | 41~80 | DHI 30mL+WM | WM | 15 d | ①⑤ |
| Wu,2012 | 46 | 48 | 27/19 | 28/20 | 64.8±11.7 | 65.3±12.6 | 52~80 | 54~81 | DHI 20mL+WM | WM | 14 d | ①⑦ |
| Jiang,2011 | 28 | 31 | 13/15 | 15/16 | NR | | 50~78 | 51~79 | DHI 40mL+WM | WM | 14 d | ①⑦ |
| Li 2012 | 32 | 36 | 17/15 | 20/16 | 65.2 | 63.5 | 52~76 | 51~78 | DHI 20mL+WM | WM | 14 d | ①⑥⑦ |
| Su 2012 | 37 | 38 | 21/16 | 22/16 | 63.01±9.34 | 61.58 ±9.17 | 45~80 | 42~85 | DHI 40mL+WM | WM | 14 d | ①③④⑤⑥⑦ |
| Chen 2013 | 64 | 70 | 30/34 | 37/33 | NR | | 44~78 | 43~79 | DHI 40mL+WM | WM | 14 d | ① |
| Wang 2010 | 80 | 80 | 44/36 | 46/34 | 65.75±6.86 | 66.36±5.73 | 52~78 | 55~80 | DHI 30mL+WM | WM | 14 d | ②③④⑤ |
| Gao,2011 | 30 | 32 | 16/14 | 17/15 | 65.5 | 63.2 | 45~76 | 40~79 | DHI 30mL+WM | WM | 14 d | ① |
| Han,2016 | 50 | 50 | 30/20 | 29/31 | 70.1±2.4 | 70.3±2.6 | 55~80 | 54~80 | DHI 30mL+WM | WM | 14 d | ① |
| Fang 2013 | 48 | 48 | 50/46 | | 50. 6 ± 5. 9 | | 52~64 | | DHI 20mL+WM | WM | 14 d | ①⑦ |
| Li 2017 | 40 | 40 | 43/37 | | 66.5±10.2 | | 50~77 | | DHI 30mL+WM | WM | 14 d | ①②⑦ |
| Zhong,2011 | 35 | 35 | 33/37 | | 72±5 | | 61~78 | | DHI 20mL+WM | WM | 14 d | ① |
| Fan, 2016 | 52 | 115 | 27/26 | 59/56 | 54.8±3.0 | 54.7±3.1 | 50~77 | 48~77 | DHI 20mL+WM | WM | 14 d | ①⑦ |
| Xu,2014 | 45 | 45 | 24/21 | 25/20 | 70.9±1.2 | 71.3±1.1 | 57~79 | 56~78 | DHI 20mL+WM | WM | 14 d | ① |
| Liu 2018 | 47 | 47 | 30/17 | 29/18 | 64.83±6.24 | 64.75±6.19 | 54~82 | 53~81 | DHI 30mL+WM | WM | 14 d | ①②⑦ |
| Tao,2011 | 20 | 55 | 12/8 | 34/21 | 55.4 ± 2.3 | 56.5 | 42~76 | 43~78 | DHI 30mL+WM | WM | 15 d | ①⑤⑥⑦ |
| Jiang 2016 | 54 | 54 | 32/22 | 30/24 | 60. 96 ±9.19 | 61.78 ± 8.36 | 62~76 | 53~72 | DHI 30mL+WM | WM | 14 d | ⑤⑥⑦ |
| Zhang,2015 | 50 | 50 | 26/24 | 28/22 | 58.6±9.2 | 60.3±7.4 | 45~72 | | DHI 30mL+WM | WM | 14 d | ①⑤⑥ |
| Zou 2013 | 40 | 40 | 22/18 | 21/19 | 57.9±9.8 | 58.7±10.1 | 42~75 | 41~76 | DHI 30mL+WM | WM | 14 d | ①⑦ |
| Tian, 2020 | 81 | 81 | 52/29 | 50/31 | 63.5 ± 5.2 | 63.5 ± 5.1 | 51~73 | 50~72 | DHI 20mL+WM | WM | NR | ①③④⑤⑥ |

*E, experiment group; C, control group; M, male; F, female; NR, not report; WM, western medicine; ①clinical effectiveness rate；②the activities of daily living (ADL); ③low shear blood viscosity (LBV); ④high shear blood viscosity (HBV); ⑤plasma viscosity (PV); ⑥fibrinogen (FIB); ⑦ adverse reactions (ADRs).*

**References**

1. Ru J. Effect of Honghua injection combined with edaravone on neurological function and the effect of serum IL-6, TNF-α and IL-17 levels in patients with acute cerebral infarction. The Medical Forum 2021, **25**(29)**:** 4192-4194.

2. Xiu-mei W. Clinical observation of edaravone combined with safflower injection in adjuvant treatment of acute cerebral infarction. World Latest Medicine Information 2016, **16**(53)**:** 108-112. DOI:10.3969/j.issn.1671-3141. 2016.53.077

<http://kns.cnki.net/KCMS/detail/detail.aspx?FileName=WMIA201653077&DbName=CJFQ2016>

3. Shu-jing L. Observation on the curative effect of safflower injection combined with ozagrel sodium on acute cerebral infarction. Journal of Clinical Medical Literature 2016, **3**(7)**:** 1360, 1361.

<https://d.wanfangdata.com.cn/periodical/lcyydzzz201607128>

4. Wei-ying Z. Observation on curative effect of safflower injection in treating 50 cases of acute cerebral infarction. Mod Diagn Treat 2013, **24**(17)**:** 3874-3875.

<http://kns.cnki.net/KCMS/detail/detail.aspx?FileName=XDZD201317021&DbName=CJFQ2013>

5. Jin-mei L, Xin-juan Z. Contrast Observation of Curative Effect of Honghua Injection on Cerebral Infarction. Shanxi Med J 2013, **42**(09)**:** 1030-1031.

<http://kns.cnki.net/KCMS/detail/detail.aspx?FileName=SXYY201309034&DbName=CJFQ2013>

6. Han-zhang L, Yue-peng Y, Fen-zhen S, Peng-fei S. Low molecular weight heparin sodium combined with safflower injection in treating of acute cerebral infarction. China Foreigh Medical Treatment 2012, **31**(31)**:** 89-90.

<http://kns.cnki.net/KCMS/detail/detail.aspx?FileName=HZZZ201231059&DbName=CJFQ2012>

7. Xiao-bei L. Clinical observation on 66 cases of acute cerebral infarction treated with integrated traditional Chinese and Western Medicine. Strait Pharmaceutical Journal 2012, **24**(06)**:** 104-105.

<http://kns.cnki.net/KCMS/detail/detail.aspx?FileName=HAIX201206050&DbName=CJFQ2012>

8. Jiang Z. Clinical Observation on Treatment of 68 Acute Cerebral Infarction with Honghua Injection. China Medical Engineering 2011, **19**(12)**:** 146-147.

<http://kns.cnki.net/KCMS/detail/detail.aspx?FileName=YCGC201112098&DbName=CJFQ2011>

9. Jun G. Observation of curative effect of safflower injection in auxiliary treatment of acute cerebral infarction. Chin J of Clinical Rational Drug Use 2011, **4**(16)**:** 89. <http://kns.cnki.net/KCMS/detail/detail.aspx?FileName=PLHY201116083&DbName=CJFQ2011>

10. Chao-ling L, Xia L, Bo-sheng F. Clinical Observation on 100 Cases of Acute Cerebral Infarction Treated by Honghua Injection. Chinese Journal of Practical Nervou s Diseases 2010, 13(19): 35-36.

https://kns.cnki.net/KCMS/detail/detail.aspx?FileName=HNSJ201019025&DbName=CJFQ2010

11. Wen-hui L. Clinical analysis of safflower injection in the treatment of acute cerebral infarction. Chian Modern Medicine 2010, **17**(28)**:** 51, 56.

<http://www.wanfangdata.com.cn/details/detail.do?_type=perio&id=jkr201028028>

12. Jing X, Jiu-ling S, Cheng-shou L. Safflower Injection in Treating 50 Cases of Acute Cerebral Infarction. Medical Recapitulate 2005, **11**(8)**:** 768. <https://kns.cnki.net/kcms/detail/detail.aspx?dbcode=CJFD&dbname=CJFD2005&filename=YXZS200508046>

13. Rong-ya L. Clinical Observation on Treatment of 76 Cases of Acute Cerebral Infarction with Honghua Injection. Chinese Community Doctor 2005(06)**:** 12. <http://kns.cnki.net/KCMS/detail/detail.aspx?FileName=ZGSQ200506011&DbName=CJFQ2005>

14. Sheng-jun L, De-jing T. Observation of Curative Effect of Honghua Injection on Acute Cerebral Infarction. China Academic Journal 2004, **4**(2)**:** 17. <https://kns.cnki.net/kcms/detail/detail.aspx?dbcode=CJFD&dbname=CJFD2004&filename=SZXL200402014>

15. Gui-yue S. Clinical Observation on the Treatment of Acute Cerebral Infarction with Integrated Traditional Chinese and Western Medicine. Liaoning Journal of traditional Chinese Medicine 2002, **29**(12)**:** 751.

<https://kns.cnki.net/kcms/detail/detail.aspx?dbcode=CJFD&dbname=CJFD2002&filename=LNZY200212053>

16. Qi S, Xiqing S. Observation on the effect of safflower yellow injection combined with butylphthalide sodium chloride injection in the treatment of acute cerebral infarction. shandong medical journal 2021, **61**(13)**:** 72-75. <https://kns.cnki.net/kcms/detail/detail.aspx?FileName=SDYY202113020&DbName=CJFQ2021>

17. Binyan J. Clinical effect of safflower yellow injection combined with troxerutin in the treatment of acute cerebral infarction. Overview of Medicine 2022(04)**:** 171-173. <https://kns.cnki.net/kcms/detail/detail.aspx?FileName=MGYI202204059&DbName=CJFQTEMP>

18. Jian-ping T, Pan J, Xiao-yan Y. Efficacy and safety of safflor yellow in the treatment of acute cerebral infarction. J Mod Med Health 2013, **29**(16)**:** 2524-2525.

<http://kns.cnki.net/KCMS/detail/detail.aspx?FileName=XYWS201316078&DbName=CJFQ2013>

19. Lijing C, Shu L, Junzhe Y, Jian L. Study on Effects of Safflower Yellow Injection on Hemorheology and Neurological Function in Patients with Acute Cerebral Infarction. Chinese Archives of Traditional Chinese Medicine 2019, **37**(02)**:** 484-488.

<http://kns.cnki.net/KCMS/detail/detail.aspx?FileName=ZYHS201902057&DbName=DKFX2019>

20. Hui-min G, Ze-yu L, Lijun S, Shu-fang W. Clinical observation of safflower yellow injection in the treatment of patients with acute cerebral infarction. Journal of Guangxi Medical University 2016, **33**(05)**:** 886-888. <http://kns.cnki.net/KCMS/detail/detail.aspx?FileName=GXYD201605045&DbName=CJFQ2016>

21. Zhi J, Xiu-bo F. Clinical study of Safflower Yellow for injection combined with argatroban in treatment of acute cerebral infarction. Drugs & Clinic 2016, **31**(02)**:** 158-162. <http://kns.cnki.net/KCMS/detail/detail.aspx?FileName=GWZW201602007&DbName=CJFQ2016>

22. Xiao-yong H. Clinical observation of safflor yellow combined with edaravone in the treatment of cerebral infarction. 2011, **27**(21)**:** 3294-3295. <https://kns.cnki.net/kcms/detail/detail.aspx?dbcode=CJFD&dbname=CJFD2011&filename=XYWS201121061>

23. Le-Jun L, Yu-Mei L, Ben-Yu Q, Shan J, Xin L, Hong-Ming D, Peng-Cheng H, Jiong S. The Value of Safflower Yellow Injection for the Treatment of Acute Cerebral Infarction: A Randomized Controlled Trial. EVID-BASED COMPL ALT 2015, **2015:** 1-6.

<http://www.hindawi.com/journals/ecam/2015/478793/>

24. Liang C, Yongcui M. Analysis of the application value of urinary kallidinogenase combined with Guhong injection in the treatment of acute cerebral infarction. China Foreign Medical Treatment 2022, **41**(01)**:** 92-95.

<https://kns.cnki.net/kcms/detail/detail.aspx?FileName=HZZZ202201023&DbName=CJFQ2022>

25. Yang S. Effects of defibrase combined with Guhong injection on neurological function and hemorheology in patients with acute cerebral infarction. Liaoning Journal of traditional Chinese Medicine 2021, **48**(10)**:** 140-142. <https://kns.cnki.net/kcms/detail/detail.aspx?FileName=LNZY202110038&DbName=DKFX2021>

26. Yang Z, Liang M, Qigang Z. Effects of Guhong injection combined with Salvia miltiorrhiza polyphenols on neurological function and hemorheology in patients with acute cerebral infarction. Clinical Focus 2021, **36**(03)**:** 212-215. <https://kns.cnki.net/kcms/detail/detail.aspx?FileName=LCFC202103004&DbName=CJFQ2021>

27. Shuangdong L, Juan L, Hang Y. Effect of Guhong injection combined with argatroban on acute cerebral infarction and its effect on serum SOD and MDA levels. Drug Evaluation Research 2021, **44**(03)**:** 566-570. <https://kns.cnki.net/kcms/detail/detail.aspx?FileName=YWPJ202103017&DbName=DKFX2021>

28. Jianlin X, Yanqing F. Observation on the efficacy of aspirin combined with guhong Injection in the treatment of cerebral infarction. Modern Hospital 2020, **20**(4)**:** 599-601. <http://www.wanfangdata.com.cn/details/detail.do?_type=perio&id=xdyy202004038>

29. Si-de J, Yao-bin Z, Jing X, Ming-shan T, Cheng-de P. Guhong Injection for Tr eating Acute C er ebr al Infar ction in 40 C ases. China Pharmaceuticals 2016, **25**(02)**:** 40-42. <http://kns.cnki.net/KCMS/detail/detail.aspx?FileName=YYGZ201602013&DbName=CJFQ2016>

30. Fei S. Effect of Guhong Injection Combined with Ozagrel Sodium on NIHSS Score and Hemorheology of Acute Cerebral Infarction. Chinese and Foreign Medical Research 2019, **17**(10)**:** 11-13. <http://kns.cnki.net/KCMS/detail/detail.aspx?FileName=YJZY201910005&DbName=CJFQ2019>

31. Yan L. Clinical study of Guhong injection combined with butylphthalide in the treatment of acute cerebral infarction. World Latest Medicine Information 2018, **18**(53)**:** 90-91.

<https://kns.cnki.net/kcms/detail/detail.aspx?dbcode=CJFD&dbname=CJFDLAST2018&filename=WMIA201853060>

32. Yun-peng L, Qing-song Z. Clinical study on Guhong Injection combined with butylphthalide in treatment of acute cerebral infarction. Drugs & Clinic 2018, **33**(1)**:** 41-45. <https://kns.cnki.net/kcms/detail/detail.aspx?dbcode=CJFD&dbname=CJFDLAST2018&filename=GWZW201801010>

33. Jia-le H, Ke-fei Z, Bin W. The curative effect of Guhong injection combined with western medicine in the treatment of patients with ischemic cerebral infarction and its influence on hemorheology. Chin J of Clinical Ｒational Drug Use 2019, **12**(34)**:** 64-65.

<http://kns.cnki.net/KCMS/detail/detail.aspx?FileName=PLHY201934034&DbName=CJFQ2019>

34. Bo T, Sheng-tao Y. Clinical observation of Guhong Injection combined with fasudil hydrochloride in treatment of ischemic cerebral infarction. Drugs & Clinic 2016, **31**(03)**:** 306-309.

<http://kns.cnki.net/KCMS/detail/detail.aspx?FileName=GWZW201603009&DbName=CJFQ2016>

35. Zhuo Z. Multi-center randomized and open clinical study on the efficacy and safety of Guhong Injection in patients with acute cerebral infarction. Modern Preventive Medicine 2010, **37**(22)**:** 4382-4383. <http://kns.cnki.net/KCMS/detail/detail.aspx?FileName=XDYF201022080&DbName=CJFQ2010>

36. Wenbin W. Effect of troxerutin combined with Danhong Injection on nerve function and coagulation function in patients with cerebral infarction. Medical theory and practice 2021, **34**(24)**:** 4268-4270. <https://kns.cnki.net/kcms/detail/detail.aspx?FileName=YXLL202124021&DbName=CJFQ2021>

37. Jun H. Observation on the curative effect of Danhong injection combined with monosialotetrahexose ganglioside sodium in the treatment of acute cerebral infarction. Grassroots Medical Forum 2021, 25(34): 4934-4936. <https://kns.cnki.net/kcms/detail/detail.aspx?FileName=YXLT202134018&DbName=CJFQ2021>

38. Enxing W, Junhuai Z, Xuefei C, Yingxue L. Clinical study of the Danhong injection combined with edaravone in the treatment of acute cerebral infarction. Anhui Medicine 2021, **25**(09)**:** 1881-1885.

https://kns.cnki.net/kcms/detail/detail.aspx?FileName=AHYY202109044&DbName=CJFQ2021

39. Yujie S. linical efficacy of butylphthalide injection combined with Danhong injection in the treatment of acute cerebral infarction. Chinese Practical Medicine 2021, **16**(20)**:** 4-6. https://kns.cnki.net/kcms/detail/detail.aspx?FileName=ZSSA202120002&DbName=CJFQ2021

40. Jinling Z, Haijun W, Ruiqing L, Yongjian W, Jiannan L. Effects of Danhong Injection Combined with Atorvastatin Calcium in the Treatment of Patients with Lacunar Cerebral Infarction and the Incidence of Adverse Reactions. Chinese Pharmacoeconomics 2021, **16**(07)**:** 84-86. <https://kns.cnki.net/kcms/detail/detail.aspx?FileName=ZYWA202107017&DbName=CJFQ2021>

41. Qing W. Effect of Danhong Injection on serum VEGF, MMP-9, hs CRP and coagulation function in patients with acute cerebral infarction. Modern Journal of Integrated Traditional Chinese and Western Medicine 2021 Jul, 30 (19) 2021.

<https://kns.cnki.net/kcms/detail/detail.aspx?dbcode=CJFD&dbname=CJFDLAST2021&filename=XDJH202119020&uniplatform>

42. Lei Y, Jin-ping Y. Clinical Observation on 40 Cases of Cerebral Infarction Treated with Aspirin and Danhong Injection. Chinese Journal of Ethnomedicine and Ethnopharmacy 2018, **27**(23)**:** 123-124.

<http://kns.cnki.net/KCMS/detail/detail.aspx?FileName=MZMJ201823043&DbName=CJFQ2018>

43. De-qing Y. Clinical Efficacy of Danhong Combined with Edaravone Injection in the Treatment of Acute Cerebral Infarction and Its Influence on Serum Related Factors. Chinese and Foreign Medical Research 2019, **17**(36)**:** 130-132. <http://kns.cnki.net/KCMS/detail/detail.aspx?FileName=YJZY201936056&DbName=CJFQ2019>

44. Ling-yun F, Yu Y. The Curative Effect of Danhong Injection on Acute Ischemic Stroke and Its Influence on Cerebral Blood Flow Automatic Ｒegulation Function. China Academic Journal 2019, **34**(05)**:** 670-673. <http://kns.cnki.net/KCMS/detail/detail.aspx?FileName=GMZY201905007&DbName=CJFQ2019>

45. Shui-sheng W. Clinical observation of Danhong injection combined with piracetam in the treatment of cerebral infarction. J Med Theor ＆ Prac 2019, **32**(22)**:** 3626-3628. <http://kns.cnki.net/KCMS/detail/detail.aspx?FileName=YXLL201922021&DbName=CJFQ2019>

46. Qing-hua L, Shuang-xing H, Hua-lan Y, Ying S, Zhi-lan T, Qing-guo B, Wei-zhong X, Yang C, Jun Y, Dan-hong P, Hong B. The effect of Danhong injection combined with butylphthalide injection on BDNF, PNY and NSE in patients with acute cerebral infarction. Chinese Journal of integrative medicine on cardio-cerebravascular disease, **18**(7)**:** 1148-1151. <https://kns.cnki.net/kcms/detail/detail.aspx?dbcode=CJFD&dbname=CJFDLAST2020&filename=ZYYY202007030>

47. Ling-zhi T, Juan L, Xiao-li S, Chao R, Yue WU, Ben-Yu Q. Clinical study on Danhong Injection combined with brain glycoside carnosine in treatment of acute cerebral infarction. Drugs & Clinic 2019, **34**(8)**:** 2317-2321. <http://www.wanfangdata.com.cn/details/detail.do?_type=perio&id=gwyy-zwyfc201908014>

48. Ming Z, Mi L, Yong-ping Z, Li-jun T, Shi-yu L. Effect of danhong injection combined with alprostadil on cerebral vascular reserve and neurological impairment in patients with ischemic cerebral infarction. Journal of North Sichuan Medeical College 2018, **33**(05)**:** 777-780. <http://kns.cnki.net/KCMS/detail/detail.aspx?FileName=NOTH201805037&DbName=CJFQ2018>

49. Mei-juan K, Chang-ming W, Liu Y, Sun J, Bao-chao Z. Effect of Danhong Injection and Tirofiban on Acute Cerebral Infarction and Associated NF-κB Inflammation Signal Pathway. Chinese Journal of Ｒational Drug Use 2020, **17**(05)**:** 56-60. <http://kns.cnki.net/KCMS/detail/detail.aspx?FileName=ZYYS202005015&DbName=CJFQ2020>

50. Yuling C. Application value of Danhong injection combined with edaravone in acute cerebral infarction. Digest World Latest Med Inf 2019, **19**(41).

https://kns.cnki.net/kcms/detail/detail.aspx?dbcode=CJFD&dbname=CJFDLAST2019&filename=WMIA201941111

51. Xiao-lin L. Observation on the curative effect of Danhong injection combined with edaravone in the treatment of acute cerebral infarction and its effect on hemorheology and neurological function. Chinese Journal of Integrative Medicine on Cardio-cerebrovascular Disease 2019, **17**(24)**:** 4054-4057. <http://kns.cnki.net/KCMS/detail/detail.aspx?FileName=ZYYY201924048&DbName=CJFQ2019>

52. Qing-xia L, Bo J. Observation on the curative effect of Danhong injection combined with edaravone in the treatment of acute cerebral infarction and its effect on hemorheology and neurological function. China Journal Pri Med Pharm 2020, **27**(04)**:** 423-424.

<http://kns.cnki.net/KCMS/detail/detail.aspx?FileName=ZJCY202004009&DbName=ZHYX2020>

53. Jing-wei Z, Ji-hong Q. Therapeutic effect of Danhong Injection on acute cerebral infarction and its influence on neurological function and inflammatory factors. CLIN MED 2020, **40**(02)**:** 95-97. <http://kns.cnki.net/KCMS/detail/detail.aspx?FileName=EBED202002037&DbName=CJFQ2020>

54. Juan D. Clinical efficacy of Danhong injection in patients with acute cerebral infarction and it's influence on NIHSS and ADL scores. CLIN MED 2018, **3**(18)**:** 22-23. <http://kns.cnki.net/KCMS/detail/detail.aspx?FileName=YLYS201818009&DbName=CJFQ2018>

55. Bo Q. Clinical observation on 60 cases of acute cerebral infarction treated with Danhong Injection. China Foreign Medical Treatment 2010, **29**(26)**:** 116. <http://kns.cnki.net/KCMS/detail/detail.aspx?FileName=HZZZ201026096&DbName=CJFQ2010>

56. Shi-quan Z. Clinical observation on 50 cases of acute cerebral infarction treated with aspirin enteric coated capsule and Danhong Injection. Chin J Mod Drug Appl 2009, **3**(23)**:** 113-114. <http://kns.cnki.net/KCMS/detail/detail.aspx?FileName=ZWYY200923103&DbName=CJFQ2009>

57. Yu-xing G, Ping L. Clinical observation of Ozagrel Sodium Combined with Danhong in the treatment of acute cerebral infarction. China Practical Medical 2012, **7**(35)**:** 176-178. <http://www.wanfangdata.com.cn/details/detail.do?_type=perio&id=zgsyyy201235142>

58. Zhi-ping Z. Observation on therapeutic effect of Buchang Beitong Danhong Injection on acute cerebral infarction. Practical journal of cardiac cerebral pneumal and vascular disease 2010, **18**(01)**:** 39. <http://kns.cnki.net/KCMS/detail/detail.aspx?FileName=SYXL201001024&DbName=CJFQ2010>

59. Gui-rong W, Huang H, Xiao-Li W, Quan-li Y, Hong Z. Clinical study of Danhong injection.Clopidogrel and Ozagrel Sodium in treatment of patients of acute infarction. Journal of Medical Forum 2017, **38**(05)**:** 58-60. <http://kns.cnki.net/KCMS/detail/detail.aspx?FileName=HYYX201705022&DbName=CJFQ2017>

60. Shao-yun T, Ze-long T. Clinical Study of Dan Hong Injection in the Treatment of Cerebral Infarction Caused by Branch Atheromatous Disease. Journal of Shenyang Medical College 2016, **18**(01)**:** 18-20. <http://kns.cnki.net/KCMS/detail/detail.aspx?FileName=SYYX201601011&DbName=CJFQ2016>

61. Yu-peng L, Zhen-guo W, Hao W. Danhong Injection on Acute Cerebral Infarction C-reactive Protein and Clinical Effects. Practical journal of cardiac cerebral pneumal and vascular disease 2010, **18**(10)**:** 1433-1434. <http://kns.cnki.net/KCMS/detail/detail.aspx?FileName=SYXL201010031&DbName=CJFQ2010>

62. Yong-Shang Y, Shuai W, Lei X. Changes of Hemorheology and Hemodynamics in the Cerebral Infarction Patients After Danhong Injection Therapy. Guide of China Medicine 2010, **8**(32)**:** 27-29. <http://kns.cnki.net/KCMS/detail/detail.aspx?FileName=YYXK201032016&DbName=CJFQ2010>

63. Jin-jing P, Min Z, Rui-feng Z. Effect of Danhong Injection on plasma D-dimer and platelet aggregation rate in patients with acute cerebral infarction. China Practical Medicine 2011, **6**(06)**:** 184-185. <http://kns.cnki.net/KCMS/detail/detail.aspx?FileName=ZSSA201106148&DbName=CJFQ2011>

64. Cheng Ping J, Bi-hua W, Liu F, Yi L, Gong-zhu W. Ef fects of Danhong Injection on Plasma Levels of TNF -αand IL -6 and Its Clinical Efficacy in Patients with Acute Cerebral Infarction. China Pharmacy 2008, **19**(24)**:** 1900-1902. <http://kns.cnki.net/KCMS/detail/detail.aspx?FileName=ZGYA200824035&DbName=CJFQ2008>

65. Xiao-yun W. Clinical analysis of Danhong Injection on Hemorheology in patients with acute cerebral infarction. Chinese Community Doctors 2008, **10**(02)**:** 99. <http://kns.cnki.net/KCMS/detail/detail.aspx?FileName=ZGSQ200802141&DbName=CJFQ2008>

66. Jia H, Jiang X. Effect of Danhong Injection on Hemorheology of Patients with Acute Cerebral Infarction. China Pharmacist 2008, **11**(6)**:** 691-693. <https://kns.cnki.net/kcms/detail/detail.aspx?dbcode=CJFD&dbname=CJFD2008&filename=ZYSG200806043>

67. Xue-qiong Z, Cai-hong Z, Xing-jian Z. Curative effect of Danhong injection on the patients with acute cerebral infarction and analysis of its influence on hemorheology. SH PHARMA 2015, **36**(9)**:** 25-30. <https://kns.cnki.net/kcms/detail/detail.aspx?dbcode=CJFD&dbname=CJFDLAST2015&filename=SYIY201509009>

68. Hong Y, Jia-hui L, Fang H, Bao-chao Z. Effect of Dan Hong Injection on cerebral hemodynamics and hemorheology in patients with cerebral infarction. Hainan Medical Journal 2017, **28**(7)**:** 1059-1061. <http://www.wanfangdata.com.cn/details/detail.do?_type=perio&id=hainanyx201707010>

69. Zhi-jie W. Clinical observation of Danhong Injection on 34 cases of acute cerebral infarction. Journal of New Chinese Medicine 2013, **45**(02)**:** 11-12. <http://kns.cnki.net/KCMS/detail/detail.aspx?FileName=REND201302006&DbName=CJFQ2013>

70. Yong-ping A, Chang-sheng R. The Clinical Observation of Danhong Injection Edaravone in Treatment of Acute Cerebral Infarction. Digest World Latest Med Inf 2015, **15**(01)**:** 41-75. <http://kns.cnki.net/KCMS/detail/detail.aspx?FileName=WMIA201501028&DbName=CJFQ2015>

71. Bao-zhen L. Effect of Danhong injection combined with ozagrel sodium on coagulation and blood lipid in patients with acute cerebral infarction. Modern Journal of Integrated Traditional Chinese and Western Medicine 2017, **26**(01)**:** 49-51. <http://kns.cnki.net/KCMS/detail/detail.aspx?FileName=XDJH201701017&DbName=CJFQ2017>

72. Qing-tao Z, De-qin G. Effect of Danhong Injection combined with sodium ozagrel for treating primary acute cerebral infarction. Journal of Qiqihar University of Medicine 2016, **37**(34)**:** 4315-4316. <http://kns.cnki.net/KCMS/detail/detail.aspx?FileName=QQHB201634036&DbName=CJFQ2016>

73. Yu-ping Y. Effect of Danhong injection combined with ozagrel sodium on cerebral infarction and serum homocysteine level. Proceeding o f Clinical M edicine 2013, **22**(01)**:** 22-24. <http://kns.cnki.net/KCMS/detail/detail.aspx?FileName=SXLC201301010&DbName=CJFQ2013>

74. Song G. Danhong injection combined with ozagrel sodium in the treatment of 32 cases of ischemic stroke. China Pharmaceuticals 2011, **20**(18)**:** 75-76. <http://kns.cnki.net/KCMS/detail/detail.aspx?FileName=YYGZ201118051&DbName=CJFQ2011>

75. He-yi Z. Danhong injection combined with ozagrel sodium injection in the treatment of 64 cases of acute cerebral infarction. Zhejiang Journal of Traditional Chinese Medicine 2012, **47**(5)**:** 327. <https://kns.cnki.net/kcms/detail/detail.aspx?dbcode=CJFD&dbname=CJFD2012&filename=ZJZZ201205013>

76. Cai-yun H. Clinical evaluation of Danhong injection combined with low molecular weight heparin calcium in the treatment of cerebral infarction. Capital Medicine 2017, **24**(16)**:** 67-68. <http://kns.cnki.net/KCMS/detail/detail.aspx?FileName=YYSD201716049&DbName=CJFQ2017>

77. Zhan-yuan L. Clinical observation of danhong injection combined with buflomedil for the treatment of acute cerebr. Chinese Community Doctors 2014, **30**(03)**:** 64-66. <http://kns.cnki.net/KCMS/detail/detail.aspx?FileName=XCYS201403042&DbName=CJFQ2014>

78. Ming-hui W, An-lai J, Ya-zhou C. Danhong injection combined with buflomedil in the treatment of 30 cases of acute cerebral infarction. National Medical Frontiers of China 2010, **5**(22)**:** 68. <http://kns.cnki.net/KCMS/detail/detail.aspx?FileName=YLQY201022046&DbName=CJFQ2010>

79. Shi-jiang M. Effect of Danhong injection combined with clopidogrel on Hemorheology in patients with ischemic stroke. Cardiovascular Disease Journal of integrated traditional 2017, **5**(24)**:** 100-101. <http://kns.cnki.net/KCMS/detail/detail.aspx?FileName=ZXJH201724081&DbName=CJFQ2017>

80. Xu G, Jun L. The clinical efficacy of Danhong injection combined with edaravone in the treatment of patients with acute ischemic stroke and its effect on vascular endothelial function and hemorheology. IMHGN 2017, **23**(2)**:** 236-239. <https://kns.cnki.net/kcms/detail/detail.aspx?dbcode=CJFD&dbname=CJFDZHYX&filename=GJYW201702032>

81. Xi-yue M, Zhong-jiang N, Lei N, Yun-jia Y. Efficacy Observation of Danhong Injection Combined with Edaravone in the Treatment of Acute Cerebral Infarction.China Pharmacy 2012, **23:** 23. <https://kns.cnki.net/kcms/detail/detail.aspx?dbcode=CJFD&dbname=CJFD2012&filename=ZGYA201223035>

82. Jia X. Clinical efficacy of Danhong injection combined with edaravone in the treatment of acute cerebral infarction. Chin J of Clinical Ｒational Drug Use 2016, **9**(10)**:** 113-115. <https://kns.cnki.net/kcms/detail/detail.aspx?dbcode=CJFD&dbname=CJFDLAST2016&filename=PLHY201630053>

83. Ying-wei Y, Ai-min Z, Ya-mei L, Da-zhi G. Clinical Effect of Danhong Injection Combined with Edaravone in Treating Patients with Acute Cerebral Infarction. Practical Journal of cardio cerebrovascular diseases 2017, **25**(11)**:** 83-85. <https://kns.cnki.net/kcms/detail/detail.aspx?dbcode=CJFD&dbname=CJFDLAST2018&filename=SYXL201711025>

84. Wei Z. The clinical study of Danhong injection combined with edaravone in the treatment of acute ischemic stroke. Chin J Prim Med Pharm 2017, **24**(7)**:** 1050-1053. <http://www.wanfangdata.com.cn/details/detail.do?_type=perio&id=zgjcyy201707023>

85. Yan Z, Jia-long L, De-sheng C. Effect of Danhong injection combined with edaravone injection on neurological function in patients with cerebral infarction. Drugs and Clinic 2016, **13**(17)**:** 45-46. <http://kns.cnki.net/KCMS/detail/detail.aspx?FileName=YPPJ201617014&DbName=CJFQ2016>

86. Jun-jie M. Clinical observation on the treatment of 58 cases of acute cerebral infarction with Danhong injection combined with Edaravone injection. Int J Trad Chin Med 2010, **32**(6)**:** 507-508. <https://kns.cnki.net/kcms/detail/detail.aspx?dbcode=CJFD&dbname=CJFDZHYX&filename=GWZY201006018>

87. Xi-tao Z. Curative effect observation of Danhong injection combined with edaravone injection in the treatment. China Prac Med 2015, **10**(18)**:** 36-37. <https://kns.cnki.net/KXReader/Detail?TIMESTAMP=637468402482685546&DBCODE=CJFD&TABLEName=CJFDLAST2015&FileName=ZSSA201518018>

88. Hui-min L. Clinical Observation on Danhong Injection in Treating Acute Cerebral Infarction. China Journal of Chinese Medicine 2011, **26**(10)**:** 1234-1235. <http://kns.cnki.net/KCMS/detail/detail.aspx?FileName=HNZK201110037&DbName=CJFQ2011>

89. Xin-hua S. Danhong injection in the treatment of 43 cases of acute cerebral infarction. Journal of Xianning University 2010, **24**(4)**:** 304-305. <https://kns.cnki.net/kcms/detail/detail.aspx?dbcode=CJFD&dbname=CJFD2010&filename=XNYB201004015>

90. Chun-shui Z, Yun H, Yong L, Zhi-xin F, Hui-min C. Danhong injection in the treatment of 96 cases of acute cerebral infarction. Shaanxi Journal of Traditional Chinese Medicine 2010, **31**(06)**:** 671-672. <http://kns.cnki.net/KCMS/detail/detail.aspx?FileName=SXZY201006016&DbName=CJFQ2010>

91. Gao-xiang C, Wen-e Z, Qiang W. Danhong injection in the treatment of 120 cases of acute cerebral infarction. Zhejiang Journal of Traditional Chinese Medicine 2009, **44**(3)**:** 229. <https://kns.cnki.net/kcms/detail/detail.aspx?dbcode=CJFD&dbname=CJFD2009&filename=ZJZZ200903064>

92. Bao-zhong W, Zhong-jie H. Clinical observation of DanHong Injection on acute infarction in 120 patients. National Medical Frontiers of China 2012, **7**(08)**:** 49-50. <http://kns.cnki.net/KCMS/detail/detail.aspx?FileName=YLQY201208030&DbName=CJFQ2012>

93. Wei-dan Z, Ji-feng W. Clinical observation of the short-term therapeutic effect of Dan Hong injection in the treatment of acute cerebral infarction. Modern Chinese Medicine Application 2011, **5**(06)**:** 14-15. <http://kns.cnki.net/KCMS/detail/detail.aspx?FileName=ZWYY201106009&DbName=CJFQ2011>

94. Xin C. Clinical Observation of Danhong Injection on Acute Cerebral Infarction. Med J of Communications 2008, **22**(3)**:** 266-267. <https://kns.cnki.net/kcms/detail/detail.aspx?dbcode=CJFD&dbname=CJFD2008&filename=JTYX200803027>

95. Wen-sheng H. Clinical observation of Danhong injection in the treatment of acute cerebral infarction. Journal of Guangxi Medical University 2010, **27**(6)**:** 897-898. <https://kns.cnki.net/kcms/detail/detail.aspx?dbcode=CJFD&dbname=CJFD2010&filename=GXYD201006029>

96. Yi-jie Z. Clinical observation of Danhong injection in the treatment of acute cerebral infarction. Jilin Medical Journal 2007, **28**(4)**:** 464-465. <https://kns.cnki.net/kcms/detail/detail.aspx?dbcode=CJFD&dbname=CJFD2007&filename=JLYX200704021>

97. Wen-ping F, Xue-lian H, Feng-ying M, Meng X, Qi-hua W, Ai-min Z. Clinical Efficacy of Danhong Injection in the Treatment of Acute Cerebral Infarction. Progress in Modern Biomedicine 2018, **18**(9)**:** 1772-1775. <http://www.wanfangdata.com.cn/details/detail.do?_type=perio&id=swcx201809037>

98. Hai-yan L. Clinical Effect of Danhong Injection on Acute Cerebral Infarction and the Impact on Oxidative Stress Ｒesponse. Practical Journal of cardio cerebrovascular diseases 2017, **25**(06)**:** 153-155. <http://kns.cnki.net/KCMS/detail/detail.aspx?FileName=SYXL201706049&DbName=CJFQ2017>

99 Li L. Clinical evaluation of Danhong injection in the treatment of acute cerebral infarction. World Latest Medicine Information 2015(80)**:** 142, 145. <http://www.wanfangdata.com.cn/details/detail.do?_type=perio&id=sjzxyy-e201580100>

100. Xiang-ming Z, Cai-nai Q, Yuan Y, Shui-jiang S. Clinical study of Danhong injection in the treatment of acute cerebral infarction. Journal of cardio cerebrovascular disease of integrated traditional Chinese and Western Medicine 2012, **10**(5)**:** 557. 89. <https://kns.cnki.net/KXReader/Detail?TIMESTAMP=637468446652714843&DBCODE=CJFD&TABLEName=CJFD2012&FileName=ZYYY201205026>

101. Li-jun Y. Clinical observation of Danhong injection in the treatment of acute cerebral infarction. Journal of Changchun University of Traditional Chinese Medicine 2012, **28**(2)**:** 308-309. <https://kns.cnki.net/kcms/detail/detail.aspx?dbcode=CJFD&dbname=CJFD2012&filename=CZXX201202065>

102. Hao W. Clinical observation of Danhong injection in the treatment of acute cerebral infarction. Linchuangyushijian 2012, **9**(20)**:** 38-39.

<https://kns.cnki.net/kcms/detail/detail.aspx?dbcode=CJFD&dbname=CJFD2012&filename=CZXX201202065>

103. Xiao-feng J. Danhong injection in the treatment of 31 cases of acute cerebral infarction. Shaanxi Journal of Traditional Chinese Medicine 2011, **32**(10)**:** 1301-1302. <http://kns.cnki.net/KCMS/detail/detail.aspx?FileName=SXZY201110020&DbName=CJFQ2011>

104. Hao L, Lei Z, Xiao-ping Y. Clinical Observation of Danhong injection treating cerebral infarction. Chin J of Clinical Rational Drug Use 2012, **5**(6B)**:** 5-6. <https://kns.cnki.net/kcms/detail/detail.aspx?dbcode=CJFD&dbname=CJFD2012&filename=PLHY201217006>

105. Xian-cai S, Shu-guang L, Jian-you L. Danhong injection in the treatment of 38 cases of cerebral infarction. Guagnxi Medical Journal 2012, **34**(11)**:** 1543-1545.

<http://www.wanfangdata.com.cn/details/detail.do?_type=perio&id=gxyx201211042>

106. Feng C, Ze-rui X. Danhong injection in the treatment of 143 cases of cerebral infarction. Chinese Medicine Modern Distance Education of China 2013, **11**(03)**:** 24-25. <http://kns.cnki.net/KCMS/detail/detail.aspx?FileName=ZZYY201303021&DbName=CJFQ2013>

107. Zhao-yi W, Hua H. Clinical observation of Danhong injection in the treatment of 80 cases of acute cerebral infarction. Guiding Journal of Traditional Chinese Medicine and Pharmacy 2010, **16**(09)**:** 36-37. <http://kns.cnki.net/KCMS/detail/detail.aspx?FileName=HNZB201009016&DbName=CJFQ2010>

108. Song G. Effect of Danhong Injection on acute cerebral infarction. Zhejiang Journal of Integrated Traditional Chinese and Western Medicine 2011, **21**(5)**:** 319-320. <http://www.wanfangdata.com.cn/details/detail.do?_type=perio&id=zjzxyjhzz201105013>

109. Shao-jie H. Clinical evaluation of Danhong injection combined with edaravone in the treatment of acute cerebral infarction. Journal of Clinical Medica 2016, **3**(51)**:** 10227. <http://kns.cnki.net/KCMS/detail/detail.aspx?FileName=LCWX201651119&DbName=CJFQ2016>

110. Haowei F, Xiaoyun H, Zhizhong M, Yihong H, Lin H, Yingli Y. Clinical observation of Colin Bay combined with Danhong injection on acute cerebral infarction in 48 cases. Chinese journal of ethnomedicine and ethnopharmacy 2013, **22**(07)**:** 54-55. <http://kns.cnki.net/KCMS/detail/detail.aspx?FileName=MZMJ201307037&DbName=CJFQ2013>

111. Yong-xian L, Yi-liang L, Shi-ling Z, Hua W, De C. Clinical efficacy of the edaravone combined with Danhong injection in the treatment of acute cerebral infarction and its influence on serum cytokine level. Journal of Guangdong Medical University 2017, **35**(05)**:** 469-471. <http://kns.cnki.net/KCMS/detail/detail.aspx?FileName=GDYY201705003&DbName=CJFQ2017>

112. Cheng-yun Z. Edaravone combined with Danhong injection in the treatment of acute cerebral infarction. China Medicine and Pharmacy 2011, **01**(21)**:** 66, 84.

<http://www.wanfangdata.com.cn/details/detail.do?_type=perio&id=zgyykx201121036>

113. Cun-xiu F. Evaluation of Clinical Effect of Edaravone Combined with Danhong Injection in the Treatment of Acute Cerebral Infarction. Journal of Mathematical Medicine 2016, **29**(11)**:** 1650-1651, 1652. <http://www.wanfangdata.com.cn/details/detail.do?_type=perio&id=slyyxzz201611033>

114. Jie X. Clinical analysis of edaravone combined with Danhong injection in the treatment of elderly patients with acute cerebral infarction. China Continuing Medical Education 2014, **6**(03)**:** 71-72. <http://kns.cnki.net/KCMS/detail/detail.aspx?FileName=JXUY201403046&DbName=CJFQ2014>

115. Hang-bo L, Xiao-dong G. Effect of combination of edaravone and Danhong injection in the treatment of acute cerebral infarction. Clinical Research and Practice 2018, **10**(3)**:** 21-22. <https://kns.cnki.net/KXReader/Detail?RESULT&TIMESTAMP=637468535663857421&DBCODE=CJFD&TABLEName=CJFDLAST2018&FileName=YLYS201810009>

116. Wen-biao T. Application of Danhong Injection in Treating Acute Cerebral Infarction. Heilongjiang Medical Journal 2011, **35**(08)**:** 602-604. <http://kns.cnki.net/KCMS/detail/detail.aspx?FileName=HLYX201108018&DbName=CJFQ2011>

117. Bang-zhi J, Yu-mao L, Xiu-qing C, Li-zhen H. Clinical effects of vinpocetine combined with Danhong injection for patients with acute cerebral infarction and its impact on hemorheology. Chinese Youjiang Medical Journal 2016, **44**(01)**:** 59-62. <http://kns.cnki.net/KCMS/detail/detail.aspx?FileName=YJYX201601020&DbName=CJFQ2016>

118. Hua-yu Z. Efficacy of vinpocetine combined with danhong injection and its impact on neurologic function in patients with acute cerebral infarction. INTERNAL MED 2015, **10**(05)**:** 623-625. <http://kns.cnki.net/KCMS/detail/detail.aspx?FileName=NKYT201505009&DbName=CJFQ2015>

119. Li-hua Z, Mei-xiao L, Hui L, Xiao-dan C. Clinical study of vinpocetine combined with Danhong injection in the treatment of acute cerebral infarction. Chinese Journal of Information on Traditional Chinese Medicine 2013, **20**(12)**:** 67-68. <http://kns.cnki.net/KCMS/detail/detail.aspx?FileName=XXYY201312027&DbName=CJFQ2013>

120. Zhi-qiang T, Gui-lan K. Clinical efficacy of Danhong injection combined with butylphthalide in the treatment of senile cerebral infarction. Chin J of Clinical Rational Drug Use 2020, **13**(33)**:** 4-6. <http://kns.cnki.net/KCMS/detail/detail.aspx?FileName=PLHY202033002&DbName=CJFQ2020>
