# Supplementary material for: Comparative efficacy of Honghua class injections for treating acute ischemic stroke: A Bayesian network meta-analysis of randomized controlled trials
Source: Front Pharmacol. 2022 Sep 28;13:1010533. doi: 10.3389/fphar.2022.1010533 (PMC9554475; doi:10.3389/fphar.2022.1010533)
Supplement: Supplementary file 1 [file DataSheet1.zip › Supplementary file/Supplementary Material S1.doc]

**Supplementary Material S1:** Detailed informations on included Honghua-class injections (HCIs).

| **HCIs** | **Source** | **Species / Raw materials** | **Phytochemical compositions molecular formula** | **Therapeutic claims in TCM** | **Indications** | **Quality control reported? (Y/N)** |
| --- | --- | --- | --- | --- | --- | --- |
| HI | Shanxi Zhendong Anxin biopharmaceutical Co., Ltd | Honghua (*Asteraceae, Carthamus, Carthamus tinctorius L.*) | SAFFLOWER YELLOW (C_43_H_42_O_22_);  Carthamone (C_21_H_20_O_11_);  4',5,7,8-TETRAHYDROXYFLAVANONE (C_15_H_12_O_6_) | Promoting blood circulation and removing blood stasis. | Occlusive cerebrovascular disease, coronary heart disease, vasculitis | Y - National Food and Drug Administration National Drug Standards WS_3_-B-3825-98-2012 |
| SYI | Zhejiang Yongning Pharmaceutical Co., Ltd | Safflower yellow | SAFFLOWER YELLOW(C_43_H_42_O_22_) | Promoting blood circulation and removing blood stasis | Coronary heart disease, stroke | Y - National Food and Drug Administration National Drug Standards YBZ21772005-2009Z |
| GHI | Tonghua Guhong Pharmaceutical Co., Ltd | aceglutamide and Honghua (*Asteraceae, Carthamus, Carthamus tinctorius L.*) | ACEGLUTAMIDE (C_7_H_12_N_2_O_4_)  Hydroxysafflor yellow A (C_27_H_32_O_16_) | Promoting blood circulation and removing blood stasis | Cerebrovascular diseases | Y - National Food and Drug Administration National Drug Standards  WS-10001（HD-1506）-2004-2012 |
| DHI | Buchang Pharmaceutical Co., Ltd. | Danshen *[Salvia miltiorrhiza Bunge (Lamiaceae; Salviae miltiorrhizae radix et rhizome)]* and Honghua (*Asteraceae, Carthamus, Carthamus tinctorius L.*); 2:1 | Danshensu (C_9_H_10_O_5_);  SALVIANOLIC ACID B (C_36_H_30_O_16_); PROTOCATECHUIC ALDEHYDE (C_7_H_6_O_3_);  TANSHINONE IIA (C_19_H_18_O_3_);  Rosmarinic acid (C_18_H_16_O_8_);  PROTAMINE SULFATE (H_2_O_4_S);  Hydroxysafflor yellow A(C27H32O16) | Promoting blood circulation and removing blood stasis | Ischemic cardio cerebrovascular disease | Y - National Food and Drug Administration National Drug Standards WS-11220(ZD-1220)-2002-2017Z |

HCIs: Honghua injection (HI), Safflower Yellow injection (SYI), Guhong injection (GHI) and Danhong injection (DHI)
