# Supplementary material for: Comparative efficacy of Honghua class injections for treating acute ischemic stroke: A Bayesian network meta-analysis of randomized controlled trials
Source: Front Pharmacol. 2022 Sep 28;13:1010533. doi: 10.3389/fphar.2022.1010533 (PMC9554475; doi:10.3389/fphar.2022.1010533)
Supplement: Supplementary file 1 [file DataSheet1.zip › Supplementary file/Supplementary Material S3.doc]

**Supplementary Material S3:** Search strategy for network meta-analysis.

**PubMed**

#1 "acute ischemic stroke" [MeSH Terms]

#2 "Acute Ischemic Stroke" [Title/Abstract] OR "Ischemic Stroke" [Title/Abstract] OR "Acute Cerebral Infarction" [Title/Abstract] OR "Cerebral Infarction" [Title/Abstract] OR "Brain Infarction" [Title/Abstract]

#3 #1 OR #2

#4 "Honghua Injection" [Title/Abstract] OR " Safflower Injection" [Title/Abstract]

#5 "Safflower Yellow Injection" [Title/Abstract]

#6 "Guhong Injection" [Title/Abstract]

#7 "Danhong Injection" [Title/Abstract]

#8 #4 OR #5 OR #6 OR #7

#9 randomized controlled trial [Publication Type]

#10 controlled clinical trial [Publication Type]

#11 randomized [Title/Abstract]

#12 placebo [Title/Abstract]

#13 randomly [Title/Abstract]

#14 trial [Title/Abstract]

#15 groups [Title/Abstract]

#16 "drug therapy" [Subheading]

#17 #9 OR #10 OR #11 OR #12 OR #13 OR #14

#18 animals [MeSH Terms]

#19 humans [MeSH Terms]

#20 #18 NOT #19

#21 #17 NOT #20

#22 #3 AND #8 AND #21

**Cochrane Library**

#1 acute ischemic stroke [Title Abstract Keyword]

#2 Acute Ischemic Stroke [Title Abstract Keyword]

#3 Ischemic Stroke [Title Abstract Keyword]

#4 Acute Cerebral Infarction [Title Abstract Keyword]

#5 Cerebral Infarction [Title Abstract Keyword]

#6 Brain Infarction [Title Abstract Keyword]

#7 #1 OR #2 OR #3 OR #4 OR #5 OR #6

#8 Honghua Injection [Title Abstract Keyword]

#9 Safflower Injection [Title Abstract Keyword]

#10 Safflower Yellow Injection [Title Abstract Keyword]

#11 Guhong Injection [Title Abstract Keyword]

#12 Danhong Injection [Title Abstract Keyword]

#13 #8 OR #9 OR #10 OR #11 OR #12

#14 randomized controlled trial [Title Abstract Keyword]

#15 controlled clinical trial [Title Abstract Keyword]

#16 randomized [Title Abstract Keyword]

#17 placebo [Title Abstract Keyword]

#18 randomly [Title Abstract Keyword]

#19 trial [Title Abstract Keyword]

#20 groups [Title Abstract Keyword]

#21 "drug therapy" [Subheading]

#22 #9 OR #14 OR #15 OR #16 OR #17 OR #18 OR #19 OR #20 OR #21
